# Dynamics of fault motion and the origin of contrasting tectonic style between Earth and Venus

**DOI:** 10.1038/s41598-018-30174-6

**Published:** 2018-08-08

**Authors:** Shun-ichiro Karato, Sylvain Barbot

**Affiliations:** 1 Yale University, Department of Geology & Geophysics, New Haven, CT USA; 2 Earth Observatory of Singapore, Nanyang Technological University, Singapore, Singapore

## Abstract

Plate tectonics is one mode of mantle convection that occurs when the surface layer (the lithosphere) is relatively weak. When plate tectonics operates on a terrestrial planet, substantial exchange of materials occurs between planetary interior and its surface. This is likely a key in maintaining the habitable environment on a planet. Therefore it is essential to understand under which conditions plate tectonics operates on a terrestrial planet. One of the puzzling observations in this context is the fact that plate tectonics occurs on Earth but not on Venus despite their similar size and composition. Factors such as the difference in water content or in grain-size have been invoked, but these models cannot easily explain the contrasting tectonic styles between Earth and Venus. We propose that strong dynamic weakening in friction is a key factor. Fast unstable fault motion is found in cool Earth, while slow and stable fault motion characterizes hot Venus, leading to substantial dynamic weakening on Earth but not on Venus. Consequently, the tectonic plates are weak on Earth allowing for their subduction, while the strong plates on Venus promote the stagnant lid regime of mantle convection.

## Introduction

**Figure 1:**
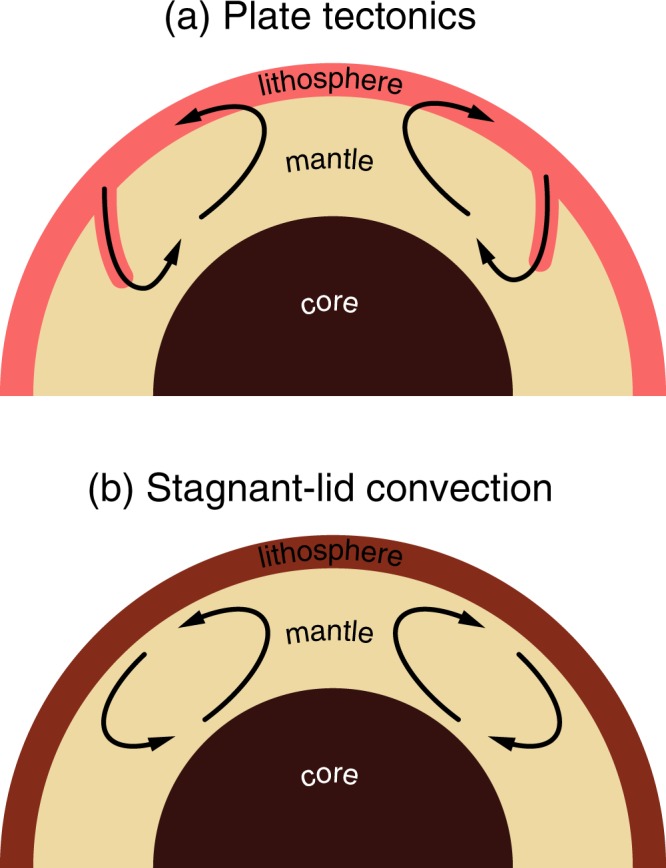
Two modes of mantle convection. When the surface layer (the lithosphere) is relatively weak, subduction occurs and plate tectonics will operate (**a**). In contrast, when the surface layer is strong, subduction cannot be initiated, and the stagnant lid mode of convection will occur (**b**) where the surface layer is stagnant and there is no large-scale materials circulation. Tectonics of Venus is considered to stagnant lid convection at least for the last ~500 Myrs^4^. The threshold strength of the lithosphere between these two regimes is ~100 MPa depending on the details of mass distribution such as the heterogeneity of the crustal thickness^4,23,24,26^.

Earth and Venus have similar size, density, and chemical composition. Consequently, one might expect that both planets evolved in a similar way. However, these planets show markedly different tectonic styles in addition to different surface temperatures and atmospheric composition. Although there are rich topographical observations on Venus showing wide-spread short-wavelength (~10s km) deformation similar to the Tibetan plateau on Earth^1,2^, the distribution of crater density shows nearly homogenous ages (~500 Myrs) of the surface. This implies that these short wavelength deformation features were formed at ~500 Myrs or before and that there is little or no large-scale materials exchange between the surface and the interior expected from plate tectonics at least for the last ~500 Myr^3^. It is generally considered that the stagnant-lid style of convection operates on Venus while plate tectonics operates on Earth^4^ (Fig. 1) implying that the surface layer (the lithosphere) on Venus is substantially stronger than that on Earth.

The topography-geoid correlation provides another constraint on the strength of the lithosphere. On Earth, topography and geoid have poor correlation for the scale of 100s-1000s km that is interpreted to imply shallow compensation (thin lithosphere)^5^. In contrast, topography and geoid have strong correlation on Venus at a similar scale, and this can be interpreted by a thick lithosphere if the variation in the crustal thickness is caused in the past (~500 Myrs ago) as suggested by the crater density distribution^4,6^. Similarly, based on the observations on surface topography on Earth (East African rift) and Venus (Beta Regio), Foster and Nimmo^7^ concluded that the faults of Venus are stronger than those of Earth.

The Venusian atmosphere is much hotter than that of Earth and is made largely of carbon dioxide^8^. Consequently, temperatures in the near surface layer of Venus are higher than those of Earth. The strength of rocks in the ductile regime decreases with temperature^9,10^. Indeed, extensive short-wavelength ductile deformation (folding) on Venus can be attributed to high near surface temperatures (not only caused by the high atmospheric temperature but also caused by the transport of hot materials some ~500 Myrs ago) (e.g.^11,12^). But as reviewed before, other observations such as the topography-gravity correlation over 100s to 1000s km scale suggest that the lithosphere on Venus is thicker than that of Earth implying that the deep lithosphere of Venus is stronger than that of Earth. It is this puzzle that we focus our attention on in this paper.

One popular idea to explain these paradoxical observations is to connect them through the loss of water (e.g.^13^): high temperature of Venus was likely caused by a slightly higher initial surface temperature than Earth’s that led to a runaway instability that resulted in a hot atmosphere promoting further loss of water^14^. The loss of water leads to a strong lithosphere preventing plate tectonics from occurring^13,15^. Another idea is that a high surface temperature leads to extensive grain-growth, making the lithosphere strong^16,17^. In both cases, a key issue is to explain why the oceanic lithosphere on Earth is weak but the lithosphere of Venus is strong.

However, when the basics of materials science of deformation and the geological observations are reviewed, it becomes clear that both of these models have some fundamental difficulties. For example, even if the oceanic lithosphere on Earth is covered with water, the main parts of the oceanic lithosphere are dry^18,19^. There have been some models to suggest relatively deep penetration of water into the oceanic lithosphere^20^, but most of these models assume that subduction is already happening and therefore these models do not explain how subduction initiates. Similarly, there are several fundamental issues for the grain-size (or “damage”) model including the fact that exceedingly small grain-size is needed to obtain sufficiently weak lithosphere and that grain-size reduction is difficult if the lithosphere is initially strong as will be discussed later^21^.

Before evaluating the plausibility of these models, let us first review the results of geodynamic modeling^22^. Geodynamic studies show that if the lithosphere is too strong, then it will stay at the surface, and the “stagnant lid” style of convection will operate^23^ (Fig. 1). The lithosphere must be weak enough for plate tectonics to start and be maintained. When the resistance against deformation of the lithosphere is characterized by the average strength (critical stress to deform a rock at a given strain-rate) for a given thickness, the threshold strength for plate tectonics to be initiated is ~100 MPa^24^ (within a factor of 2) depending on the distribution of mass at the surface^25^, corresponding to a friction coefficient of ~0.1^26^. So any model must explain why the strength of the lithosphere of Venus is higher than this critical value but that of Earth is smaller than or comparable to this value.

More precisely, in order for plate tectonics style of convection to occur, the lithosphere should be strong at most places but it must loose its strength locally and temporarily at plate boundaries to reduce the average strength to on the order of ~100 MPa or less. In this paper, we will investigate how such a mechanical behavior of the lithosphere is possible on Earth but not on Venus.

**Figure 2:**
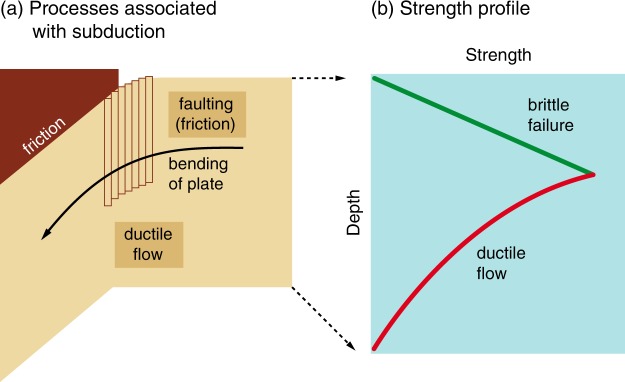
Schematic diagrams showing (**a**) the processes associated with subduction of the oceanic lithosphere and (**b**) a corresponding strength profile. (**a**) Subduction of the oceanic lithosphere needs to overcome frictional resistance against an overlying lithosphere, resistance for bending of the oceanic lithosphere by brittle failure (faulting) in the shallow region and by ductile flow in the deep region. (**b**) A schematic strength profile corresponding to the processes shown in (**a**) (for a more detailed model, see Fig. 3)

In all models on the presence or absence of plate tectonics, a key issue is the subduction of the (oceanic) lithosphere. Plate tectonic occurs only when subduction is possible^27,28^. The types of resistance against subduction are schematically shown in Fig. 2. They include friction between the subducting lithosphere and the overlying materials, and the resistance against bending of the lithosphere itself by brittle failure (faulting) or ductile flow.

The focus of this paper is to investigate the strength of the lithosphere itself to understand how the initiation of subduction is possible on Earth but not on Venus. In the next sections, we lay out a critical review of the existing models that explain the difference of convection style between the two planets. We then discuss the importance of strong weakening by shear heating during earthquakes for this debate. We describe the thermal conditions under which frictional instabilities can develop. Finally, we show how the dynamics of fault slip with strong weakening is compatible with low seismic stress drops, large rock yield stress, but overall a low strength of the oceanic lithosphere on Earth.

## Results

### Sensitivity of the strength profile on various factors

**Figure 3:**
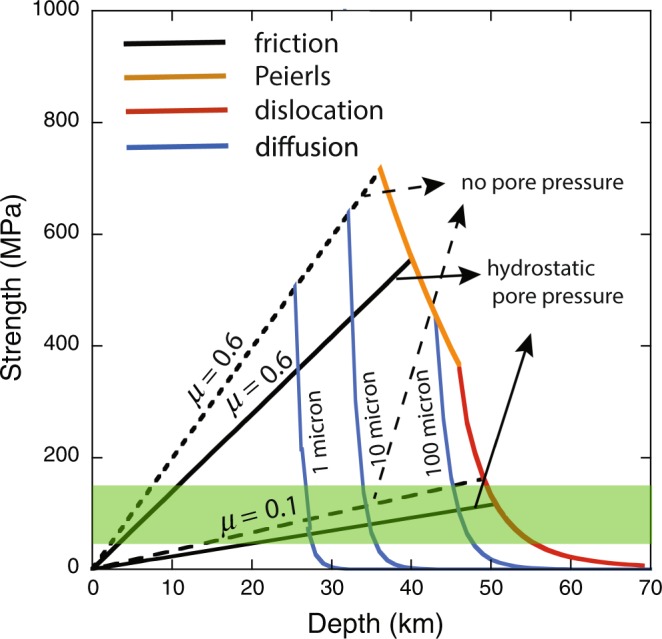
The strength profile of the lithosphere. Temperature-depth profile corresponding to 60 Myrs old oceanic lithosphere is used. Differential stress (*σ*_1_ *−* *σ*_3_) (*σ*_1_: the maximum compressional stress, *σ*_3_: the minimum compressional stress) needed for deformation at 10^−14^ s^−1^ strain-rate is plotted (shear stress is given by $\tau =\frac{{\sigma }_{1}-{\sigma }_{3}}{2}$). In the shallow part, the strength is controlled by friction (*μ*: friction coefficient), and in the deep part by plastic flow. We consider diffusion creep (numbers correspond to grain size in micron), (power law) dislocation creep and the Peierls mechanism (low-temperature plasticity). Grain-size reduction in the plastic flow regime reduces the strength, but even for extremely small grain size (1 micron), the shallow lithosphere is strong if the friction coefficient is large (0.6). The reduction of friction coefficient (to ~0.1) is an efficient way to reduce the strength of the lithosphere. The stress level below which plate tectonic would occur is shown by the green hatched region. (For the data source, see Supplementary Information).

In order to illustrate a few key points, let us first consider the strength-depth profile based on the results of rock mechanics studies. To illustrate the influence of various factors (other than temperature), we calculate the strength profile for the thermal model of the 60 Myrs old oceanic geotherm on Earth (Fig. 3) (the influence of the temperature gradient and of the surface temperature will be explored later).

Such models are based on the idea that the resistance to deformation in the shallow part is controlled by the resistance for sliding on pre-existing faults, whereas in the deeper part it is controlled by plastic deformation^29,30^. In the deep region, where the strength is controlled by plastic deformation, the strength is sensitive to rock type, strain-rate, temperature and grain-size (the effect of pressure is only moderate for a typical activation volume of 10 cc/mol^31^). We will assume that the lithosphere of both Earth and Venus is made of peridotite and use the experimental results on dry olivine based on the results suggesting relatively dry oceanic lithosphere^19,32^ (for Venusian lithosphere where the crust is thick (~30 km^11^) we use the dry flow law of diabase^12^. For the oceanic lithosphere on Earth, the contribution from the crust to the lithosphere strength is negligible).

We consider three deformation mechanisms: (i) diffusion creep, (ii) power-law dislocation creep and (iii) the Peierls mechanism (low-temperature plasticity). Grain-size of typical upper mantle rocks is several mm^33,34^ (for the processes by which grain-size is determined, see^35^). However, in shear zones, much smaller grain-size are observed typically ~ 10s of microns but in some cases down to a few microns^36,37^. Therefore grain-sizes of 1, 10, 100 micron as well as 5 mm are assumed. For a grain-size of 5 mm, the dominant mechanism of deformation is either power-law dislocation creep or the Peierls mechanism that is insensitive to grain-size.

In the shallow region where the strength is controlled by the resistance for motion of pre-existing faults, we used the following relation^38^,

1 $$\tau =\mu ({\sigma }_{n}-{P}_{pore})$$

where $\tau$ is the shear stress needed for fault motion, *μ* is the friction coefficient, *σ*_*n*_ is the normal stress, and *P*_*pore*_ is the pressure of pore fluid. We consider the strength corresponding to normal faulting that is relevant to plate bending near the trench.

To illustrate the range of strength one can get for different assumptions, several cases will be considered. For the friction coefficient, a canonical value is ~0.6. This is based on a large number of experimental studies that show that the static friction coefficient is nearly independent of rock type (including serpentinite), sliding velocity (for small sliding velocities, <0.1 m/sec)^39,40^ and temperature (to T < 600 °C; influence of shear heating is small for less than ~0.1 m/sec^41^). However, we also show the result for a friction coefficient of 0.1 for comparison.

As to the pore pressure, we consider two cases (i) zero pore pressure (no fluid on the fault plane) and (ii) pore pressure = hydrostatic pressure of water. The latter is a case where the fault is filled with water that is connected to the surface. As can be seen in Fig. 3, the influence of pore pressure is small as far as it is up to the hydrostatic pressure. In contrast, the influence of friction coefficient is large, if there are mechanisms to change the friction coefficient.

### Difficulties in reducing the strength in the ductile regime

Another important point of Fig. 3 is the fact that the degree to which small grain-size reduces the strength is limited. This latter point may need an elaboration because there have been many publications where grain-size effects were considered to play a key role in controlling the strength of the lithosphere. For example, based on the geological observations, weakening due to grain-size reduction is often proposed to explain shear localization^36,37^. Also detailed theoretical models have been developed where the main mechanism for weakening of the lithosphere is grain-size reduction by dynamic recrystallization^22,42^.

One of the obvious difficulties of the grain-size (“damage”) model is the fact that because the temperature in the shallow lithosphere is so low, one would need exceedingly small grain-size (sub-micron) to make the lithosphere weak enough (see Fig. 3). Geological observations show that the grain-size of upper mantle rocks is typically a few mm^33,34^. In very rare cases, grain-size of a few microns (not sub-microns) is observed but these are only in very thin layers (~1 cm thickness)^36,43^ (in these cases, the local strain rate will be much higher than the average strain rate, and the influence of fine grains on the average strength of the lithosphere will be limited).

More fundamentally, it is difficult to produce small grains by dynamic recrystallization under small ambient strain rates^36^, as grain-size reduction requires substantial plastic strain (~10% or more strain)^9,21,44^. If the lithosphere were strong to begin with, not much plastic strain can be produced and small grains would not form. For example, assuming 100 MPa stress, the strain in olivine at 20 km depth in the oceanic lithosphere (P = 0.7 GPa, T = 300–500 °C) in 100 Myrs would be ~10^−16^–10^−6^, too small for dynamic recrystallization to occur. Extremely small grains (a few microns) found in some pseudotachylites are formed by high local stress associated with faulting^36^, not by purely plastic deformation. Furthermore, the degree to which small grain-size affects the strength of a plate is unclear because the distribution of weak regions, for example, the spacing of shear zones, is not well defined in the previous models (see a discussion presented in^45^). We conclude that although grain-size reduction does often lead to shear localization as seen many mylonites on the continents^36,37^, the degree to which this makes the oceanic lithosphere weak and initiate subduction is limited.

Recently Kumamoto *et al*.^46^ invoked a “size effect” and proposed that the strength of olivine in the low-temperature plasticity regime might be substantially weaker than previously thought. This would reduce the strength in this regime somewhat, but Kumamoto *et al*.^46^ recognized that this effect is not enough to reduce the strength to ~100 MPa. However, the physical basis for this “size effect” is unclear as discussed in Supplementary Information. Furthermore, if this were the cause for the weakening of lithosphere on Earth, it would be difficult to explain why Venusian lithosphere is so strong because the strength in this regime decreases with temperature. Consequently, the “size effect” is not included in our model.

### Reduction in friction coefficient by high velocity fault motion

Compared to reducing the strength in the ductile regime, it would be much more effective to reduce the brittle strength by either reducing the friction coefficient or by increasing the pore pressure beyond the hydrostatic pressure^47^ (Fig. 3). Because the pore pressure in excess of hydrostatic pressure is most likely caused by heating^47^, essentially it is heating that is responsible for the reduction in frictional resistance.

Recent experimental studies showed that the friction coefficient can be substantially reduced when the velocity of fault motion exceeds a threshold value of *V*_*C*_ ~ 1 m/s^41,48,49^ (in the laboratory where the normal stress is ~10 MPa). But this reduction in friction coefficient does not occur instantaneously. At high velocities, the friction coefficient evolves with the sliding distance (*x*) from the initial static friction coefficient, ${\mu }_{o}$, to a reduced value, ${\mu }_{\infty }$, after a slip greater than the characteristic slip distance, *D*_*th*_, viz.^41^,

2 $$\mu (x)={\mu }_{\infty }+({\mu }_{o}-{\mu }_{\infty }){e}^{-\tfrac{x}{{D}_{th}}}.$$

Using this relation, it can be shown that if the total slip distance (*D*) far exceeds the critical distance for thermal weakening (*D*_*th*_), the effective friction coefficient defined by ${\int }_{o}^{D}\mu (x)\cdot {\sigma }_{n}\cdot S\cdot dx\equiv {\mu }_{eff}{\int }_{o}^{D}{\sigma }_{n}\cdot S\cdot dx$ (*S*: area of the fault, *x*: displacement) is reduced to ${\mu }_{\infty }$ (~0.1 or less^41^), viz.,

3 $${\mu }_{eff}={\mu }_{\infty }+\tfrac{{D}_{th}}{D}({\mu }_{0}-{\mu }_{\infty })(1-{e}^{-\tfrac{D}{{D}_{th}}})\Rightarrow \,{\mu }_{\infty }\,as\,D\gg {D}_{th}.$$

Because the integral defined above is the work done by friction, this definition implies that with this small effective friction coefficient, the conditions for plate tectonics is satisfied from the energetics point of view.

The causes for strong weakening at high-velocity fault motion are not fully understood, but they may involve several mechanisms. In some cases, melt is observed on the fault when friction coefficient is reduced substantially (e.g.^48^). However, weakening may not always involve melting. Some thermally activated processes such as decarbonation or dehydration reactions producing nano-size particles or high-pressure fluids might play some role^41,50,51^. However, in all these cases, high temperature from shear heating causes a reduction in the frictional strength. We, therefore, call these processes collectively as “thermal weakening”.

Thermal weakening occurs when the velocity of frictional sliding exceeds a threshold value (*V* > *V*_*C*_, *V*: velocity of sliding, *V*_*C*_: threshold velocity for thermal weakening). The threshold velocity for thermal weakening is on the order of 1 m/s^41,48^ for typical experimental conditions where the normal stress is ~10 MPa^52^. Another important condition for thermal weakening is a large enough slip distance, *D* ≫ *D*_*th*_. When these two conditions are met, and if ${\mu }_{\infty }$ is smaller than ~0.1, then thermal weakening would lead to substantial reduction in the resistance for plate subduction and would allow plate tectonics to occur.

Since all laboratory results used here are obtained at low normal stress (<~40 MPa), applications of these results to friction in the deep lithosphere where the normal stress is ~1,000 MPa or more require some scaling analysis. The scaling analysis summarized in Supplementary Information shows that thermal weakening is enhanced at higher normal stress and the conditions for *V*_*C*_ and *D*_*th*_ are likely met for friction in the deep lithosphere, and the friction coefficient (${\mu }_{\infty }$) is substantially lower than the static friction coefficient (${\mu }_{o}$), particularly at high confining pressures. This is essentially due to the fact that at a high normal stress, more work is done by friction ((work/unit area) = (normal stress) × (displacement)).

At a greater depth, the style of deformation changes from localized brittle behavior to distributed ductile one, and in the latter regime, intense shear heating will not occur. The distribution of seismicity in the old oceanic lithosphere (e.g.^53^) shows that localized deformation continues to ~50 km depth, and therefore our model will work to that depth.

Let us now consider under which conditions fast fault motion could occur. The velocity of fault motion includes a wide range^54^. A fault starts to move with a slow rate (~10^−9^ m/s), but when fault motion is unstable, the sliding velocity will be accelerated to a high value. Velocity of fault motion associated with a typical earthquake is ~1 m/s (e.g.^55^). Thermal weakening occurs only at the high end of slip rate (>~1 m/s) corresponding to regular earthquakes. However, this high-speed fault motion spontaneously occurs only when fault motion is unstable and accelerated.

**Figure 4:**
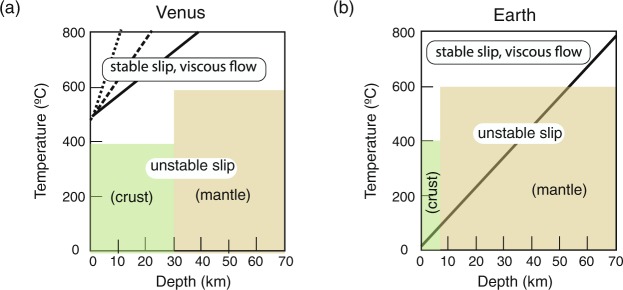
Temperature profiles (solid and broken lines) for (**a**) Venus and (**b**) Earth compared with the conditions for unstable fast fault motion (hatched regions). On Earth, the oceanic geotherm for 60 Myr old ocean is assumed. For Venus, three models of temperature-depth profiles are shown (dT/dz = 6, 12, 18 K/km). The conditions for unstable fast fault motion leading to a small effective friction coefficient are met at depths shallower than ~50 km in Earth (this depth depends on age). On Venus, the temperature in all regions exceeds the threshold temperature for unstable fast fault motion. Consequently, the strength in the shallow regions of Venus is characterized by the static friction coefficient of ~0.6, leading to a high strength that would not allow plate tectonics to occur.

Experimental studies show that unstable, fast fault motion occurs under relatively low temperatures, below ~400 °C for crustal rocks^56^ and below ~600 °C for mantle rocks^57^. The latter agrees well with the maximum depth of intra-plate earthquakes in the oceanic lithosphere^57^. Given these conditions, it is clear that on Venus, the conditions for unstable fast fault motion are not met, but they are met in the shallow regions of Earth’s lithosphere (Fig. 4). This leads to a small effective friction coefficient on Earth, but not on Venus.

### Strength profiles for Earth and Venus

**Figure 5:**
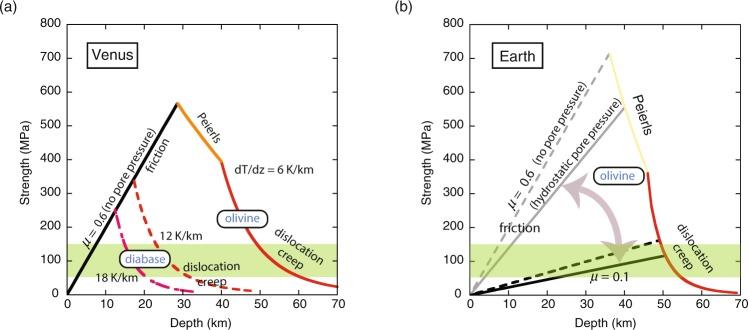
The strength profiles of (**a**) Venus and (**b**) Earth. For Venus, three temperature profiles dT/dz = 6, 12, 18 K/km are considered and the pore pressure was assumed to be zero. The lowest temperature gradient (dT/dz = 6 K/km) corresponds to much of the recent temperature profiles^11^. Higher temperature gradients (dT/dz = 12, 18 K/km) would represent the period soon after the large-scale over-turn. Venus has a much higher surface temperature than Earth leading to a higher friction coefficient (0.6) and hence a higher strength in the shallow part. For Earth, the oceanic geotherm corresponding to the age of 60 Myr is used. Both zero pore pressure and hydrostatic pore pressure are considered (same as Fig. 3). The strength of Earth’s lithosphere is heterogeneous and evolves with time due to thermal weakening caused by unstable fast fault motion (Fig. 6): initial static high friction to dynamic low friction. The effective (average) strength corresponds to a dynamic low friction coefficient (see equation (3); for the details on calculating the strength for evolving friction, see Supplementary Information).

Assuming a small effective friction coefficient for Earth (0.1) and a large one for Venus (0.6), we calculated the strength profiles for these two planets (Fig. 5**)**. For Earth, the strength of the oceanic lithosphere (with an age of 60 Myrs) is calculated. To account for a possible effect of pore pressure, we consider two cases, one is a case where the fault is filled with water that has the hydrostatic pressure (the fault rocks has the lithostatic pressure), and another is a case of no pore pressure. A key feature of the strength profile of Earth’s lithosphere is that because fault motion is unstable, the brittle strength is not constant but evolves. At a static condition the friction coefficient is high (${\mu }_{o}$ ~ 0.6), but it evolves to a low value (${\mu }_{\infty }$ ~ 0.1 or less at high pressure) when slip velocity is high and slip distance far exceeds *D*_*th*_.

The link between the reduced friction by thermal weakening and the average strength of the lithosphere depends on the entire history of stress evolution during the seismic cycle. A key issue in this connection is that the static stress drop associated with earthquakes is substantially smaller (~10 MPa or less) than the peak stress associated with static friction (~100 MPa or higher). As far as we accept this, we conclude that the strength in the brittle regime in Earth is approximately represented by the profile corresponding to $\mu \simeq {\mu }_{\infty }=$ 0.1, and with this friction coefficient, Earth’s lithosphere is weak enough for plate tectonics to operate.

On Venus, the pore pressure is assumed to be zero, and three possible temperature-depth profile models are considered (dT/dz = 6, 12, 18 K/km). The small temperature gradient model is preferred based on the analysis of thermal structures based on Venusian deformation based partly on the results of laboratory data on dry diabase deformation by Mackwell *et al*.^11,12^, but we also use the higher temperature gradient that would correspond to the period soon after the large scale over-turn^12^.

The lithosphere strength for Venus exceeds the critical strength for plate tectonics. In contrast, the calculated strength for Earth is compatible with plate tectonics.

## Discussion

### The threshold strength of the lithosphere for plate tectonics

The preceding discussions are based on the presumption that the strength of the lithosphere needs to be ~100 MPa (within a factor of 2) or less for plate tectonics to operate. This presumption is based on the results of a large number of numerical modelings^4,16,23–26,58^. We note, however, that Buffett and Becker^59^ suggested that subduction could continue with the standard strength model of the lithosphere (average the strength of ~500 MPa or more). The difference between these two sets of studies may be caused by the fact that the initiation of subduction is more difficult than maintaining subduction. Once subduction has started, a large negative buoyancy force is available making it easier to maintain it. Consequently, we believe that the threshold strength of the lithosphere of ~100 MPa (within a factor of 2) is appropriate in investigating whether plate tectonics operates or not for a given planet.

### The role of water (or hydrous minerals)

An alternative model for the weak lithosphere on Earth is the role of hydrous minerals. Indeed, some hydrous minerals (e.g., talc) reduce the friction coefficient^60^, and low friction coefficients are reported for samples from the San Andreas fault^61^ and from the fault in the Japan trench where the 2011 Mw 9.1 Tohoku earthquake took place^62^. However, it is not clear if a substantial amount of hydrous minerals is available in the deep oceanic lithosphere (~20–40 km depth) where plate bending must occur to initiate subduction. The oceanic lithosphere is depleted with water and other volatiles^63^, and it is difficult to imagine the presence of hydrous minerals in the deep lithosphere (for more details, see Supplementary Information). Furthermore, unlike talc or smectite, the hydrous minerals that would be present in the mantle portion of the lithosphere such as serpentine have a friction coefficient not much different from other materials below ~400 °C^64^. We conclude that strong weakening from fast, unstable fault slip is a more likely mechanism to reduce the strength of tectonic plates.

### A comparison to other observations

How does our model explain other observations? The largest strike-slip earthquake sequence of the 2012 Mw 8.6 Indian Ocean earthquake occurred on near-perpendicular conjugate faults, indicating a low effective friction coefficient^65^. The Indian Ocean earthquake cut the entire lithosphere, showing that rupture processes may reduce fault strength deep into the lithosphere. Therefore this observation suggests that the dynamic friction coefficient in most of the oceanic lithosphere is small.

**Figure 6:**
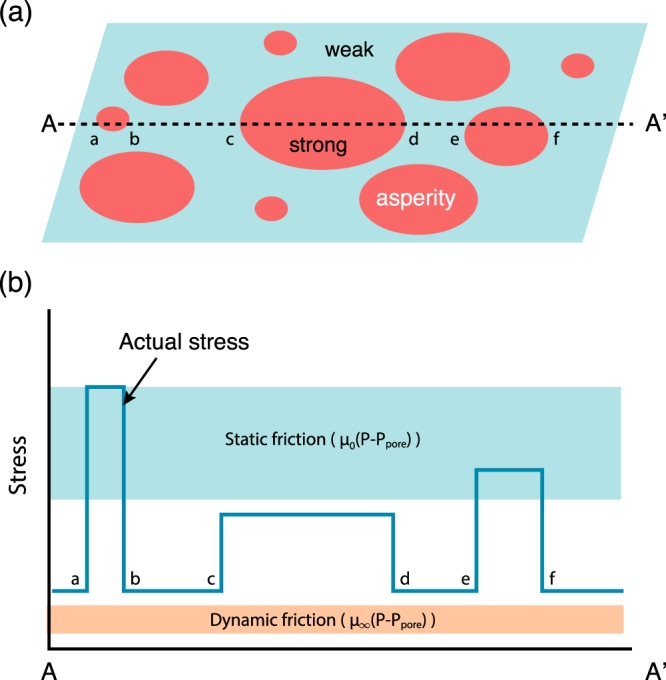
A model of a fault plane and corresponding stress distribution. (**a**) A fault plane is made of weak part (green background) and strong parts (red regions (large asperities)). (**b**) A large asperity initially deforms elastically when weak regions creep or slide and stress at a large asperity increases with time until the local stress reaches the critical stress for the asperity to break. The critical stress to break an asperity is approximately the same as the stress corresponding to static friction and depends nearly linearly on depth (this is why the stress corresponding to “static friction” has a broad range). Stress at a given point on a fault is also expected to be time dependent. After the break of an asperity, this region becomes weak (due to shear weakening) and stress is re-distributed (Figs S2, S3). If strong enough dynamic weakening is activated in regions of large static strength, the resulting long-term strength may be at the dynamic level. As a result, the stress may be at the static level (blue curve) most of the time, but at the dynamic level (purple band) for most of the fault slip.

However, such a model raises an issue of how to explain the initiation of fault slip on the strong asperity controlled by static friction because the strength corresponding to the static friction coefficient exceeds the tectonic stress level. Also, if the static and dynamic friction coefficients are so different, one may also ask how to explain inferred low stress drop (~10 MPa or less) from the analyses of earthquake mechanisms^66^. We believe that a key to solve this question is the heterogeneity of strength on a fault (Fig. 6) as discussed by Rice^47^ and others^67,68^.

A fault contains various regions with different strength (an asperity model^69^). Fault motion occurs in weak regions under the tectonic stress that leads to stress concentration in the strong regions. This eventually breaks a strong asperity. When slip occurs in an unstable manner, stress concentration on a strong asperity occurs rapidly when the propagating slip front reaches close to the strong asperity. The strength threshold is reached dynamically, leading to a large dynamic (peak-to-peak) stress drop, but a low static (before minus after the seismic event) stress drop. Such a behavior leads to a low slip-averaged stress while still compatible with small seismic stress drops^67,70^. We performed a numerical modeling of stress evolution in such a heterogeneous fault, and show that for a certain choice of parameters characterizing the fault properties, we can reproduce the fault behavior that is consistent with low static stress drop and high static strength, i.e., a high time-averaged stress coeval with a low slip-averaged stress (for details see Supplementary Information).

**Figure 7:**
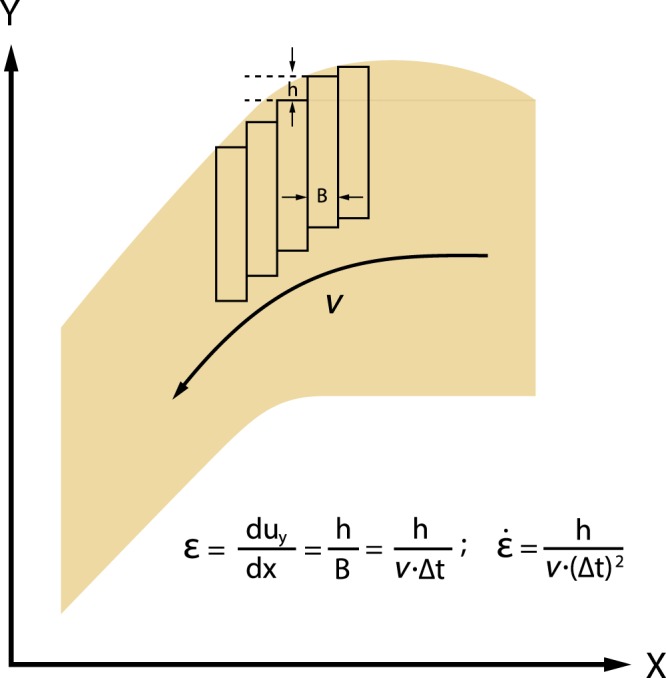
A diagram showing a relation between bending strain and faulting in a bending plate. *h*: vertical displacement associated with a normal fault, *B*: the mean spatial interval of faults, *v*: velocity of plate motion, ${\rm{\Delta }}t$: mean time interval of normal fault earthquakes

Are there enough earthquakes near the trench to accommodate deformation that occurs during subduction? Shear strain of an oceanic lithosphere caused by faulting is given by $\varepsilon =\tfrac{h}{B}$ (see Fig. 7) where *h* is the displacement associated with faulting, *B* is the mean spacing of normal fault near trenches (${B}={\upsilon }\cdot {\rm{\Delta }}{t}$ where *v* is the velocity of plate motion and ${\rm{\Delta }}t$ is the mean interval of earthquakes associated with these faults). Then the strain rate caused by these faults is $\dot{\varepsilon }=\tfrac{{h}}{{\upsilon }\cdot {({\rm{\Delta }}{t})}^{2}}$. Given a typical value of *h* ~1 m estimated from that for 1933 M = 8.2 Sanriku earthquake^71^, and the plate velocity of 10^−9^ m/s, we should have Δ*t* ≈ 10^4^ year for this mechanism to make strain rate of 10^−14^ s^−1^ (strain rate associated with plate bending at a trench). Chapple and Forsyth^72^ estimated the frequency of normal fault earthquakes near trenches for the whole Earth, and found that those with magnitude 8 occur every ~30 years. Given the total length of trench of ~50,000 km, and assuming a typical length of normal faults along the trench of ~100 km (corresponding to a M (magnitude) = 8 earthquake^73^), this can be translated to the mean time interval of Δ*t* ≈ 10^4^ years. This result agrees well with the present model.

### Summary and perspectives

Our model provides a possible explanation for the operation (initiation) of plate tectonics on Earth but not on Venus. Indeed, there are several observations on Venus that suggest a high strength of the near-surface layer compared to that of Earth^7^ including the positive correlation between topography and gravity field^74^, lack of subduction for ~500 Myrs^3^. However, the causes for the different style of convection (stagnant lid convection) on Venus are not entirely clear. In addition to the strong faults, other factors may also contribute to the lack of plate tectonics on Venus such as the absence of a low viscosity asthenosphere^75^ and the presence of a thick, buoyant crust^11^.

Also, although evidence of large-scale tectonics such as plate tectonics is lacking on Venus, Venus shows evidence of extensive short-wavelength (~10s of km scale) deformation shown by widespread distribution of folding (e.g.^1,2,76^). There are several models to explain these short-wavelength tectonic features on Venus^2,76^. However, most of these features are old (~500 Myrs), and therefore one would need to consider different thermal structures than the current one. For example, soon after a large-scale over-turn at ~500 Myrs ago, near surface temperature would be substantially higher than the current temperatures that might have facilitated small-scale deformation (this would corresponding to the strength profile for a higher dT/dz in Fig. 5a).

The implications of the strength of the lithosphere on the global dynamics and thermal evolution of a planet remain unclear. Moresi and Solomatov^26^ discussed that even if the strength of the near-surface layer plays an important role, the net heat loss and hence the thermal evolution of a planet is still controlled by mantle flow. In contrast, Conrad and Hager^77^ argued that the lithosphere strength controls thermal evolution (see also^28,78^).

Our model suggests that the strength of faults plays a key role in controlling not only the nature of near-surface tectonics but also the global dynamics, such as the style of mantle convection. However, the nature of friction is not well understood at high normal stress relevant to faulting in the deep lithosphere (~20–30 km depth). Experimental studies on high velocity friction need to be extended to higher normal stress conditions. Also, the dynamics of heterogeneous fault needs further detailed studies including a broad range of parameters characterizing the slip behavior.

Since the effective strength of faults depends on near-surface temperature, the coupling between climate and internal dynamics may be important in analyzing the geological evolution of planets (e.g.^16^). Finally, we point out that our proposed model predicts the absence of large quakes on Venus.

## Materials and Methods

### Strength of rocks

Strength of rocks in both brittle and ductile regimes is calculated using the standard definition of the strength in these two regimes. Strength is the differential stress needed to deform a rock at a given strain rate. We choose a strain rate of 10^−14^ s^−1^ appropriate for deformation near a trench.

For the ductile regime, we consider three deformation mechanisms, i.e., power-law dislocation creep, the Peierls mechanism (low-temperature plasticity) and diffusion creep. In the ductile regime, the strength depends on materials. We assume olivine-rich rocks for the mantle of both planets. The oceanic crust makes little contribution to ductile strength on Earth, but for Venus thick crust (~30 km) makes some contributions. We assumed basaltic rocks (e.g., diabase) for the Venusian crust.

For the brittle regime, we assume that many faults exist and the strength is controlled by the stress needed to move the pre-existing faults. However, the resistance against the fault motion is not constant when the velocity of fault motion becomes high enough caused by unstable fault slip (unstable flip occurs at low temperature on Earth but not at high temperature on Venus). In these cases, the lithosphere strength in the brittle regime evolves with time and both the initial and final friction coefficients are used to calculate the strength in the brittle regime.

The details of used constitutive relations and the data source are given in Supplementary Information.

### Earth and Venus structure

For Earth, 7 km thick oceanic crust and underlying upper mantle (made mainly of olivine) is assumed. For Venus, 30 km crust and upper mantle below is assumed based on a model by Nimmo and McKenzie^11^. A strength-depth profile also depends on the temperature-depth profile. We use a model oceanic geotherm corresponding to the age of 60 Myr for Earth.

Temperature-depth relation in Venus is not well constrained. The crater density observations suggest that there was a large-scale over-turn of materials from the interior to the surface at ~500 Myrs ago, after that there was no major large-scale tectonics on Venus. Soon after the large-scale over-turn, the surface temperature was high whereas after the over-turn thermal gradient is likely reduced because cold materials are brought into the deep interior. Based on the review by Nimmo and McKenzie^11^, we use a model with dT/dz = 6 K/km (*T*: temperature, *z*: depth) for a representative thermal gradient, but we also use a higher gradient, dT/dz = 12 and 18 K/km to explore the strength profile corresponding to the period in which shallow regions are hotter.

Uncertainties in these models are discussed in Supplementary Information.

### Data availability

All data needed to evaluate the conclusions in the paper are present in the paper and/or the Supplementary Information. Additional data related to this paper may be requested from the authors.

## Electronic supplementary material

Supplementary Information

## References

[1] Solomon SC, Head JW (1991). Fundamental issues in the geology and geophysics of Venus. Science.

[2] Brown CD, Grimm RE (1999). Recent tectonic and lithospheric thermal evolution in Venus. Icarus.

[3] Schaber GG (1992). Geology and distribution of impact craters on Venus: What are they telling us?. Journal of Geophysical Research.

[4] Solomatov VS, Moresi LN (1996). Stagnant lid convection on Venus. Journal of Geophysical Research.

[5] Watts, A. B. *Isostasy and Flexure of the Lithosphere*. (Cambridge University Press, 2001).

[6] Simons M, Hager BH, Solomon SC (1994). Global variations in the geoid/topography admittance of Venus. Science.

[7] Foster A, Nimmo F (1996). Comparisons between the rift systems of East Africa, Earth and Beta Regio, Venus. Earth and Planetary Science Letters.

[8] Schubert G (1980). Structure and circulation of the Venus atmosphere. Journal of Geophysical Research.

[9] Poirier, J.-P. *Creep of Crystals*. (Cambridge University Press, 1985).

[10] Karato, S. *Deformation of Earth Materials: Introduction to the Rheology of the Solid Earth*. (Cambridge University Press, 2008).

[11] Nimmo F, McKenzie D (1998). Volcanism and tectonics on Venus. Annual Review of Earth and Planetary Sciences.

[12] Mackwell SJ, Zimmerman ME, Kohlstedt DL (1998). High-temperature deformation of dry diabase with application to tectonics on Venus. Journal of Geophysical Research.

[13] Kaula WM (1994). The tectonics of Venus. Philosophical Transaction of the Royal Society of London A.

[14] Hartmann, W. K. *Moons and Planets*. 4th edition edn, (Wadsworth Publishing Company, 1999).

[15] Kaula, W. M. Venus: A contrast in evolution to Earth. *Science* **247** (1990). 10.1126/science.247.4947.1191 17809275

[16] Foley BJ, Bercovici D, Landuyt W (2012). The conditions for plate tectonics on super-Earths: Inference from convection models with damage. Earth and Planetary Science Letters.

[17] Bercovici D, Ricard Y (2014). Plate tectonics, damage and ingeritance. Nature.

[18] Peslier AH, Bizimis M (2015). Water in Hawaiian peridotites: A case for a dry metasomatized oceanic mantle lithosphere. Geochemistry, Geophysics, Geosystems.

[19] Hirth G, Kohlstedt DL (1996). Water in the oceanic upper mantle - implications for rheology, melt extraction and the evolution of the lithosphere. Earth and Planetary Science Letters.

[20] Faccenda M, Gerya TV, Burlini L (2009). Deep slab hydration induced by bending-related variations in tectonic pressure. Nature Geoscience.

[21] Karato S, Toriumi M, Fujii T (1980). Dynamic recrystallization of olivine single crystals during high temperature creep. Geophysical Research Letters.

[22] Bercovici, D., Tackley, P. J. & Ricard, Y. In *Treatise on Geophysics* Vol. 7 (ed Schubert, G.) 271–318 (Elsevier, 2015).

[23] Solomatov VS, Moresi LN (1997). Three regimes of mantle convection with non-Newtonian viscosity and stagnant lid convection on the terrestrial planets. Geophysical Research Letters.

[24] van Heck, H. J. & Tackley, P. J. Planform of self-consistently generated plates in 3D spherical geometry. *Geophysical Research Letters* **35**, 10.1029/2009GL035190 (2008).

[25] Lourenço D, Rozel A, Tackley PJ (2016). Melting-induced crustal production helps plate tectonics on planets. Earth and Planetary Science Letters.

[26] Moresi L, Solomatov V (1998). Mantle convection with a brittle lithosphere: thoughts on the global tectonic styles of the Earth and Venus. Geophysical Journal International.

[27] McKenzie, D. P. In *Island Arcs, Deep Sea Trenches and Back-Arc Basins* (eds Talwani, M. & Pitman, W. C. III) 57–61 (American Geophysical Union, 1977).

[28] Korenaga J (2013). Initiation and evolution of plate tectonics on Earth: Theories and observations. Annual Review of Earth and Planetary Sciences.

[29] Goetze C, Evans B (1979). Stress and temperature in the bending lithosphere as constrained by experimental rock mechanics. Geophysical Journal of Royal Astronomical Society.

[30] Kohlstedt DL, Evans B, Mackwell SJ (1995). Strength of the lithosphere: constraints imposed by laboratory measurements. Journal of Geophysical Research.

[31] Karato S (2010). Rheology of the deep upper mantle and its implications for the preservation of the continental roots: A review. Tectonophysics.

[32] Peslier, A. H., Schönbächler, M., Busemann, H. & Karato, S. Water in the Earth’s interior: Distribution and origin. *Space Science Reviews* in press (2018).

[33] Mercier J-CC (1980). Magnitude of the continental lithospheric stresses inferred from rheomorphic petrology. Journal of Geophysical Research.

[34] Avé Lallemant HG, Mercier J-CC, Carter NL (1980). Rheology of the upper mantle: inference from peridotite xenoliths. Tectonophysics.

[35] Karato S (1984). Grain-size distribution and rheology of the upper mantle. Tectonophysics.

[36] Jin D, Karato S, Obata M (1998). Mechanisms of shear localization in the continental lithosphere: inference from the deformation microstructures of peridotites from the Ivrea zone, northern Italy. Journal of Structural Geology.

[37] Handy MR (1989). Deformation regimes and the rheological evolution of fault zones in the lithosphere: the effects of pressure, temperature, grain size and time. Tectonophysics.

[38] Paterson, M. S. & Wong, T.-F. *Experimental Rock Deformation - The Brittle Field*. (Springer, 2005).

[39] Byerlee JD (1978). Friction of rocks. Pure and Applied Geophysics.

[40] Stesky RM (1978). Mechanisms of high temperature frictional sliding in Westerly granite. Canadian Journal of Earth Sciences.

[41] Di Toro G (2011). Fault lubrication during earthquakes. Nature.

[42] Bercovici, D. & Ricard, Y. Grain-damage hysteresis and plate tectonic states. *Physics of the Earth and Planetary Interiors* **253** (2016).

[43] Obata M, Karato S (1995). Ultramafic pseudotachylyte from Balmuccia peridotite, Ivrea-Verbana zone, northern Italy. Tectonophysics.

[44] Derby B, Ashby MF (1987). On dynamic recrystallization. Scripta Metallurgica.

[45] Karato S (2014). Some remarks on the models of plate tectonics on terrestrial planets: From the view-point of mineral physics. Tectonophysics.

[46] Kumamoto KM (2017). Size effects resolve discrepancies in 40 years of work on low-temperature plasticity. Science Advances.

[47] Rice, J. R. Heating and weakening of faults during earthquake slip. *Journal of Geophysical Research* **111**, 10.1029/2005JB004006 (2006).

[48] Tsutsumi A, Shimamoto T (1997). High-velocity frictional properties of gabbro. Geophysical Research Letters.

[49] Tullis, T. E. In *Treatise on Geophysics* Vol. 4 (ed Schubert, G.) 139–159 (Elsevier, 2015).

[50] Ferri F, Di Toro G, Hirose T, Shimamoto T (2010). Evidence of thermal pressurization in high‐velocity friction experiments on smectite‐rich gouges. Terra Nova.

[51] Brantut N, Passelègue FX, Deldicque D, Rouzaud J-N, Schubnel A (2016). Dynamic weakening and amorphization in serpentine during laboratory earthquakes. Geology.

[52] Niemeijer, A. R., Di Toro, G., Nielsen, S. & Di Felice, F. Frictional melting of gabbro under extreme experimental conditions of normal stress, acceleration, and sliding velocity. *Journal of Geophysical Research* **116**, 10.1029/2010JB008181 (2011).

[53] McKenzie D, Jackson JA, Priestley K (2005). Thermal structure of oceanic and continental lithosphere. Earth and Planetary Science Letters.

[54] Beroza GC, Ide S (2011). Slow earthquakes and nonvolcanic tremor. Annual Review of Earth and Planetary Sciences.

[55] Scholz CH (1989). Mechanics of faulting. Annual Review of Earth and Planetary Sciences.

[56] Scholz CH (1998). Earthquakes and friction laws. Nature.

[57] Boettcher, M. S., Hirth, G. & Evans, B. Olivine friction at the base of oceanic seismogenic zones. *Journal of Geophysical Research* **112**, 10.1029/2006JB004301 (2007).

[58] Tackley PJ (1998). Self-consistent generation of tectonic plates in three-dimensional mantle convection. Earth and Planetary Science Letters.

[59] Buffett, B. A. & Becker, T. W. Bending stress and dissipation in subducted lithosphere. *Journal of Geophysical Research* **117**, 10.1029/2012JB009205 (2012).

[60] Hirauchi K-i, Fukushima K, Kido M, Muto J, Okamoto A (2016). Reaction-induced rheological weakening enables oceanic plate subduction. Nature Communications.

[61] Fulton, P. M. & Saffer, D. M. Potential role of mantle-derived fluids in weakening the San Andreas Fault. *Journal of Geophysical Research* **114**, 10.1029/2008JB006087 (2009).

[62] Fulton PM (2013). Low coseismic friction on the Tohoku-Oki fault determined from temperature measurements. Science.

[63] Plank T, Langmuir AH (1992). Effects of melting regime on the composition of the oceanic crust. Journal of Geophysical Research.

[64] Chernak LJ, Hirth G (2010). Deformation of antigorite serpentine at high temperature and pressure. Earth and Planetary Science Letters.

[65] Masuti S, Barbot S, Karato S, Feng L, Banerjee P (2016). Upper-mantle water stratification inferred from the observations of the 2012 Indian Ocean earthquake. Nature.

[66] Kanamori H, Anderson DL (1975). Theoretical basis of some empirical relations in seismology. Bulletin of Seismological Soceity of America.

[67] Noda, H., Dunham, E. M. & Rice, J. R. Earthquake ruptures with thermal weakening and the operation of major faults at low overall stress levels. *Journal of Geophysical Research* **114**, 10.1029/2008JB006143 (2009).

[68] Rice JR (1999). Flash heating at asperity contacs and earthquake instabilities. EOS, Transactions of American Geophysical Union.

[69] Lay T, Kanamori H, Ruff L (1982). The asperity model and the nature of large subduction zone earthquakes. Earthquake Prediction Research.

[70] Yamashita T (1976). On the dynamical process of fault motion in the presence of friction and inhomogeneous initial stress Part I. Rupture propagation. Journal of Physics of the Earth.

[71] Kanamori H (1971). Seismological evidence for a lithospheric normal faulting - The Sanriku earthquake of 1933. Physics of the Earth and Planetary Interiors.

[72] Chapple WM, Forsyth DW (1979). Earthquakes and bending of plates at trenches. Journal of Geophysical Research.

[73] Lay, T. & Wallace, T. C. *Modern Global Seismology*. (Academic Press, 1995).

[74] Solomon SC (1992). Venus tectonics: An over-view of Magellan observations. Journal of Geophysical Research.

[75] Richards, M. A., Yang, W. S., Baumgardner, J. R. & Bunge, H.-P. Role of a low-viscosity zone in stabilizing plate tectonics: Implications for comparative planetology. *Geochemistry*, *Geophysics*, *Geosystems* **2**, 10.1029/2000GC000115 (2001).

[76] Head JW (1990). Processes of crustal formation and evolution on Venus: An analysis of topography, hypsometry, and crustal thickness variation. Earth, Moon, and Planets.

[77] Conrad CP, Hager BH (1999). The thermal evolution of an Earth with strong subduction zones. Geophysical Research Letters.

[78] Korenaga, J. Energetics of mantle convection and the fate of fossil heat. *Geophysical Research Letters* **30**, 10.1029/2003GL016982 (2003).

