# Supplementary material for: Dynamics of fault motion and the origin of contrasting tectonic style between Earth and Venus
Source: Sci Rep. 2018 Aug 8;8:11884. doi: 10.1038/s41598-018-30174-6 (PMC6082836; doi:10.1038/s41598-018-30174-6)
Supplement: Supplementary file 1 — Supplementary Information [file 41598_2018_30174_MOESM1_ESM.pdf]

Supplementary Information for

Dynamics of fault motion and the origin of contrasting tectonic style between Earth and Venus

Shun-ichiro Karato and Sylvain Barbot

correspondence to: shun-ichiro.karato@yale.edu

This PDF file includes:

Supplementary Text
Figs. S1 to S3
Table S1

Supplementary Text

Strength of rocks

The strength of the lithosphere is controlled either by the stress needed to deform a rock plastically or by the stress needed to fracture a rock. In the deep lithosphere where temperature and pressure are high, the strength is controlled by plastic flow. In this regime, the stress $\sigma\left(=\sigma_1-\sigma_3\right)$ (σ_1: the maximum compressive stress, σ_3: the minimum compressive stress) needed to deform the lithosphere depends strongly on various variables as [1,2],

$$\sigma\left(=\sigma_1-\sigma_3\right)=F\left(T,P,\dot{\varepsilon},d,C_W\right) \qquad \text{(S-1)}$$

where T is temperature, P is pressure, $\dot{\varepsilon}$ is strain-rate, d is grain size and C_W is water content. The functional form, $F\left(T,P,\dot{\varepsilon},d,C_W\right)$, depends on the mechanisms of plastic deformation. Three mechanism of deformation are considered [2] and the source of the data are summarized in **Table S1**: diffusion creep, power-law dislocation creep and the low-temperature plasticity (the Peierls mechanism). To a good approximation, plastic rheology of a rock is isotropic and the strength given by equation (S-1) depends only on the magnitude of deviatoric stress ($\sigma\left(=\sigma_1-\sigma_3\right)$) and does not depend on the stress state (compression or tension). The strength depends on grain-size in diffusion creep regime, but the strength is independent of grain-size for other mechanisms.

Recently, Kumamoto et al. [3] suggested that the strength in the low-temperature plasticity may depend on the size such as grain-size or the size of the indentation in such a way that the strength corresponding to low-temperature plasticity for a large geological grain-size might be lower than previous model. However, we consider that the experimental basis for this hypothesis is not strong. For example, the data point by Druiventak et al.[4] on a coarse grained sample plays a key role in order to justify this model. However, the experimental study by Druiventak et al.[4] was conducted in the semi-brittle regime, and consequently, their strength likely under-estimates the true strength corresponding to low-temperature plasticity.

In addition, the data by Evans and Goetze[5] on a single crystal was plotted for the "size" of ~3 μm assuming that in their experiments, the relevant "size" was the size of indentation. This is highly questionable. In these micro-indentation experiments, the size of indentation is measured as a function of load, and a broad range of indentation size is explored. The results show that the hardness (strength) is independent of the load (size).

Kumamoto et al. [3] also used the "agreement" between Druiventak et al.[4] and the results of dislocation velocity measurements to support their model. The calculation of low-temperature strength from the dislocation velocity measurements involves a number of assumptions and is not straightforward. One of the important factors is the influence of the use of a very thin sample. The stress acting on a dislocation in a very thin sample includes the image stress caused by the

presence of a free surface [6], and consequently, applications of such measurements to estimate the "true" dislocation mobility are complex and the uncertainties are large.

For these reasons, we will not include the "size effects" on low-temperature plasticity suggested by Kumamoto et al. [3] (see also [7]).

The strength corresponding to diffusion creep depends strongly on grain-size. We consider the grain-size of 1, 10, 100 microns (for a discussion on grain-size see grain size section). Strength in this regime is generally sensitive to water content, but since the oceanic lithosphere is depleted with water (see Water in the oceanic lithosphere section), we use the flow law parameters for dry conditions. The results summarized in **Fig. 3** show that even an unusually small grain-size of 1 micron, the strength is still high in the shallow, low temperature regions.

In the shallow lithosphere, deformation occurs by brittle fracture. In the brittle regime, deformation is localized and occurs along the fault plane. A common assumption is that there are many pre-existing faults in the lithosphere, and the strength in this regime is controlled by the resistance against the motion of pre-existing faults.

Experimental studies show that a fault moves when the shear stress on the fault plane exceeds a critical value given by [8,9],

$$\tau = \mu\left(\sigma_n - P_{pore}\right) \qquad \text{(S-2)}$$

where σ_n is the normal stress to the fault, τ is shear stress ($\tau = \frac{\sigma_1 - \sigma_3}{2}$), μ is friction coefficient, and P_{pore} is the pore pressure (it is assumed that water fills the fault plane and is connected to the surface) and we ignored the cohesive strength.

The shear stress (τ) on the given fault plane depends on the applied stress (σ_1, σ_3) and their orientations relative to the fault plane. Consequently, the resistance for the fault motion depends on the stress state: fault motion is more difficult for the compressional stress state (τ_{thrust} : thrust fault) than for the tensional stress state (τ_{normal} : normal fault) ($\tau_{transform}$: transform fault is in between). A simple model shows [10],

$$\tau_{normal} = \frac{\mu}{\sqrt{\mu^2+1}+\mu}\left(P - P_{pore}\right). \qquad \text{(S-3a)}$$

$$\tau_{thrust} = \frac{\mu}{\sqrt{\mu^2+1}-\mu}\left(P - P_{pore}\right) \qquad \text{(S-3b)}$$

$$\tau_{tranform} = \frac{\mu}{\sqrt{\mu^2+1}}\left(P - P_{pore}\right) \qquad \text{(S-3c)}$$

where *P* is the (lithostatic) pressure, and hence, $\tau_{thrust} > \tau_{transform} > \tau_{normal}$. The higher value of strength for compression (thrust fault) than those for tension (normal fault) implies that it is more difficult to initiate subduction than to maintain subduction.

Equation (S-2) does not specify the velocity at which sliding occurs. As far as the shear stress on a fault exceeds the value given by equation (S-2), sliding starts. Sliding will start with the velocity imposed by the geological boundary conditions

(i.e., slow initial velocity). In some cases, sliding is stable but in other cases sliding is unstable. For a stable slow sliding, sliding velocity is low and the friction coefficient has a nearly universal value, $\mu \approx 0.6$ [11]. However, if sliding is unstable, then the sliding velocity becomes high and the friction coefficient will be reduced to less than $\mu \approx 0.1$ [12] when sliding velocity exceeds a critical value (~ 1 m/sec).

If one uses a canonical value of static friction coefficient ($\mu = 0.6$), then the average strength of the lithosphere substantially exceeds the limit for plate tectonics (~100 MPa) although the magnitude of strength in the brittle regime depends on the stress state (τ_{thrust} versus τ_{normal}). If there are mechanisms to reduce the friction coefficient to ~0.1, then the lithosphere would be weak enough to allow plate tectonics.

<u>Grain-size</u>

Grain-size reduction is often invoked to reduce the strength of the lithosphere[13-17]. Indeed, reduced grain-size is observed in the shear zones [13,14,18]. The most plausible process to cause grain-size reduction is dynamic recrystallization, i.e., deformation-induced refinement of grain-size [19,20]. In such a case, the grain-size is primarily controlled by the differential stress [19,21] (**Fig. S1**).

Typical tectonic stress on Earth's lithosphere is 10-100 MPa [22-24] that would lead to the grain-size of 20-500 microns (typical stress in the hot asthenosphere is 0.1-1 MPa that would lead to the grain-size of 1-10 mm). Grain-size of rocks in the lithosphere is determined mainly when rocks were deformed at hot asthenospheric conditions [25]. Consequently, grain-size in most of the lithosphere is several mm [24-26]. In the typical mylonites (fine-grained rocks) in the shear zones, the grain-size of olivine is 10-100 microns [13,14,18,27].

The maximum stress in the lithosphere estimated above is based on the calculation of density contrast in the convecting mantle, namely $\sigma \approx \rho_o \cdot \alpha \cdot \Delta T \cdot g \cdot z \approx$ 100 MPa ($\alpha = 3\text{x}10^{-5}$ K^{-1}, $\rho_o = 3300$ kg/m^3, $\Delta T \approx 1000$ K, g=9.8 m/s^2, z $\approx$ 100 km). The density contrast caused by phase transformations in the subducted slab also contributes to the stress, but its magnitude can be larger than the above estimate [22,28,29].

Rocks with grain-size less than 10 micron are very rare, and found only in areas with intense collision at the later stage of deformation where stress is concentrated in a narrow zone [14,30]. Even with extremely small grain sizes (e.g., 1

micron), the strength is still large in the shallow regions where temperature is low (**Fig. 2**). Therefore we conclude that it is difficult to reduce the grain-size to the degree that the oceanic lithosphere is weak enough to bend and subduct.

Finally, we note that grain-size model for rheological weakening and our model are not necessarily exclusive. Temperature dependence of stability of fault motion emphasized in our model may indirectly be related to temperature dependence of grain-size (healing). Also, grain-size of fault gouge may also control the nature of fault creep that could result in the heterogeneity of mechanical properties of a fault (see Dynamics of and stress evolution in a heterogeneous fault).

Scaling the thermal weakening behavior

Laboratory high-speed friction experiments show a marked drop in the resistance for slip when slip velocity exceeds a critical value [12,31]. Laboratory experiments also show that this reduction in resistance occurs only beyond a certain displacement.

These laboratory experiments cover a broad range of slip velocity ranging from 10^{-8} to 10 m/s corresponding to the rate of slip for slow earthquakes to normal earthquakes and there is no need for extrapolation in terms of slip rate [32]. However, the normal stress in these studies is limited to ~40 MPa [33]. When we apply these results to the deep lithosphere, say ~30 km depth, the normal stress (confining pressure) goes to ~1000 MPa. Consequently, one needs to address the validity of applying these laboratory data to deep lithospheric conditions with particular emphasis on the influence of normal stress (confining pressure).

Three parameters play a key role in the thermal weakening: the threshold velocity (V_c) and the threshold slip distance (D_{th}) for thermal weakening, and the "steady-state (final)" friction coefficient (μ_∞). Let us address the scaling issues one by one.

Threshold velocity (V_c)

Weakening (a substantial reduction in the friction coefficient) at high velocities is likely due to temperature increase. Indeed, evidence of melting is often observed in samples where a substantial reduction in friction coefficient is observed [31,34]. However, some other processes that occur at high temperatures may also be

responsible for the reduction in the friction coefficient including some thermally activated chemical reactions that form materials reducing the friction coefficient (tribochemical reactions)[12].

In the following, we use the results by [12], and derive a scaling relationship for the critical distance D_{th} assuming that weakening occurs when temperature is high enough. When the velocity of sliding is high, large work is done on the fault plane and substantial heating will occur. This issue can be analyzed by solving a problem of temperature increase caused by the mechanical work done on the fault plane. A simple one-dimensional model predicts the temperature rise as [12],

$$(\Delta T)_c = \frac{\tau_e V_c}{\rho C_P}\sqrt{\frac{D_{th}}{\pi \kappa V_c}} \qquad \text{(S-4)}$$

where $(\Delta T)_c$ the temperature increases needed for melting ($(\Delta T)_c = T_c - T_o$ where T_c is the critical temperature for weakening and T_o is the initial temperature), τ_e is the effective stress, V_c is the threshold velocity for melting, ρ is density, C_P is specific heat, κ is thermal diffusivity and D_{th} is the threshold slip distance for thermal weakening. Using the relations $\tau_e \approx 0.64\tau_o \approx 0.4\sigma_n$ and $D_{th} = a \cdot \sigma_n^{-b} = a \cdot \sigma_n^{-1.24}$ [12], we obtain

$$(\Delta T)_c \propto \sqrt{V_c} \cdot \sigma_n^{1-\frac{b}{2}} = \sqrt{V_c} \cdot \sigma_n^{0.38} . \qquad \text{(S-5)}$$

Hence,

$$V_c \propto (\Delta T)_c^2 \cdot \sigma_n^{-0.76} \,. \qquad \text{(S-6)}$$

The critical temperature for frictional weakening is unlikely to change much with pressure in the pressure range that we consider (P<1 GPa)[35] . Therefore we conclude that the threshold velocity for thermal weakening decreases with pressure (depth), i.e., thermal weakening becomes easier with pressure (depth). This is because the amount of work done by friction increases with pressure (normal stress).

In the above model, frictional heating is assumed to occur in an infinitesimally thin layer. This approximation is valid when thermal diffusion distance ($\sqrt{\frac{\pi \kappa V_c}{D_{th}}}$) exceeds the thickness of the fault plane (*h*) where shear deformation occurs [36]. If the thickness of fault plane exceeds thermal diffusion thickness ($h > \sqrt{\frac{\pi \kappa V_c}{D_{th}}}$), then equation (S-4) should be replaced with (see [36]),

$$\Delta T = \frac{\tau_e V_c}{\rho c_P h} \,. \qquad \text{(S-7)}$$

In such a case, threshold velocity will be inversely proportional to the effective stress and hence depth. We conclude that at a deeper region where deformation is more diffuse (in the semi-brittle regime), critical velocity for thermal weakening is lower. In an extreme case where shear is very diffuse, then in a plausible effective stress, temperature rise is not enough to cause thermal

weakening. This will be the case in the deep region (~40 km in the 60 Myrs old oceanic lithosphere (see **Fig. 2**)) where the dominant mode of deformation gradually changes to ductile flow (e.g., [37]). In this study, we focus on relatively shallow lithosphere (~10-30 km), and therefore the influence of shear zone thickness will not be important.

Threshold slip distance for thermal weakening (D_{th})

The thermal weakening occurs only after a finite slip. The displacement due to the fault motion (D) must far exceed the threshold slip distance, D_{th} ($D \gg D_{th}$). Again this is due to the fact that one needs to produce a certain amount of heat to melt the rock. At a typical laboratory condition where normal stress is ~10 MPa (this corresponds to a depth of ~0.3 km), D_{th} = 2-4 m. The displacement associated with an earthquake of Mw (magnitude) = 8 is typically ~1-5 m. Therefore under these shallow conditions, the criterion for thermal weakening ($D \gg D_{th}$) is not always met.

However, the critical distance for thermal weakening decreases strongly with the normal stress (pressure), and thermal weakening will occur more easily at deeper depths. To show this, we use an empirical rule summarized by Di Toro et al. [12], viz.,

$$D_{th} = a \cdot \sigma_n^{-b} \qquad \text{(S-7)}$$

where a and b are the parameters that depend on the rock type. For all rocks b is positive (for peridotite, a=78, b=1.24 (unit for D_{th} is m, and unit for σ_n is MPa)), and this means that the threshold slip distance decreases with confining pressure. Again this is due to the fact that the mechanical work done by friction increases with confining pressure. Niemeijmer et al. [33] conducted high-velocity friction experiments to the normal stress of 40 MPa and obtained slightly different scaling law from that of equation (S-7), but both the relation (S-7) and the one obtained by Niemeijmer et al. [33] predict that shear heating is more efficient at higher normal stress and the critical distance decreases with depth.

Using this scaling, we find that for σ_n = 1,000 MPa (depth of ~30 km), D_{th} ~1 cm for peridotite. The slip distance (D) for a typical Mw=8 earthquake is on the order of 1 m (for the Sanriku earthquake of 1933, D~3 m [38]). Therefore we conclude $D \gg D_{th}$ and thermal weakening occurs easily at a depth deeper than ~10 km.

Scaling for the final friction coefficient (μ_∞)

In most of laboratory studies, the thermal weakening is characterized by the reduction in the friction coefficient from $\mu_o \approx$ 0.6-0.7 to $\mu_\infty \approx$ 0.1 [12]. Thermal weakening could involve various processes including sub-solidus mechanisms as well as melting [12]. Since melting is detected in most cases where thermal weakening is observed [12,31,34], we will consider a case where the reduction in friction coefficient is caused by melting.

The physical basis for "friction" is (nearly) point contact at the rough surface (e.g., [2,11]). One can easily derive a linear relation between shear stress and normal

stress by assuming that both are controlled by the slip rate-independent “yield strength” of a material, and such a model explains a near universal value of (static) friction coefficient that is independent of temperature (e.g., [2,11]).

However, when a melt layer is present then the physical basis for using the “friction” law becomes unclear because the resistance in this case depends on the slip velocity and the thickness of the melt layer. Nielsen et al. [34] provided a model that includes melt extrusion and temperature sensitive melt viscosity to come up with an equation for the shear stress at the final stage of fault motion as,

$$\tau_\infty = \sigma_n^{1/4}\left(\frac{A}{\sqrt{R}}\right)\sqrt{\left[log(2V/W)/(V/W)\right]} \qquad \text{(S-8)}$$

where A is a constant that depends on the latent heat etc. that is nearly independent of normal stress, R is the characteristic length of melt migration before melt goes to injection vein and W is the characteristic velocity given by $W = \sqrt{\frac{8T_c\kappa\rho C_p}{\eta}}$ (η : melt viscosity). R is considered to be the spacing of injection veins to which melt will intrude [34]. Based on the field observations it is on the order of ~10 cm, similar to the size of a laboratory sample. The term $log(2V/W)/(V/W)$ is only weakly dependent on normal stress, and if we assume that $\frac{A}{\sqrt{R}}$ and $log(2V/W)/(V/W)$ are independent of normal stress, then we obtain,

$$\mu_\infty \propto \sigma_n^{-3/4} \qquad \text{(S-9)}$$

implying that the resistance for shear decreases with normal stress (pressure). Therefore the resistance for shear after melting (μ_∞) in the deep lithosphere will be even smaller than the resistance observed in the laboratory at the lower normal stress. Niemeijmer et al. [33] obtained slightly different scaling law from that of equation (S-8) and (S-9), namely $\tau_\infty \propto \sigma_n^{0.5}$ and $\mu_\infty \propto \sigma_n^{-0.5}$, but both results predict that shear heating is more efficient at higher normal stress (at $\sigma_n = 1{,}000$ MPa, μ_∞ will be ~20-30% of μ_∞ at $\sigma_n = 40$ MPa).

The reason for the decrease in the "friction coefficient" with normal stress is that the resistance force for fault motion depends on the thickness of the melt layer that decreases weakly (less than linear) with normal stress (pressure). The relation (S-8) is supported by the experimental observation [34]. However, high-velocity friction behavior has not been studied under the deep lithospheric conditions ($\sigma_n >$ 100 MPa).

Applicability of the above model to subsolidus mechanisms of thermal weakening is not clear. It is clear, however, that any thermally activated processes likely occur more pronouncedly at higher normal stress, and consequently, it is likely that the reduction in friction coefficient at high normal stress is larger at higher normal stress.

In summary, we conclude that thermal weakening is likely more pronounced in the deep lithosphere and therefore the resistance for high velocity fault motion will be smaller in the deep lithosphere than in the laboratory experiments at low pressures. However, high velocity fault motion occurs only at relatively low

temperatures, i.e., in the relatively shallow lithosphere on Earth but nowhere on Venus.

Venus structure

The temperature of the surface of Venus is ~470 C [39]. The atmosphere has very little water [40] although isotopic observations (D/H ratio) suggest that Venus had a lot more water in the past [41].

The crust of Venus is made mainly of basaltic rocks [42]. However, the thickness of the crust is not well constrained. Head [43] estimated the crustal thickness from the wave-length of deformation features. He concluded that a majority (~75%) of Venus has low topography ("low land") where the crust is 10-20 km thick with a small fraction of regions with the thicker crust (30-50 km). There is a hint that the crustal thickness has changed with geologic time: thin crust soon after the episodic geological activities some 500 Myr ago, and after that crust has grown. However, based on the estimate of elastic thickness of the lithosphere and the revised experimental data of creep strength of dry diabase [44], Nimmo and McKenzie [45] suggested a larger crust thickness, ~30 km. We choose the crust thickness of 30 km.

There are not many constraints on the composition of the mantle. We simply assume that the mantle of Venus has the same composition of Earth. The only difference is water content. The atmosphere of Venus has very little water. And if the crust has as much water as crustal rocks on Earth, its strength would be too low to explain the topography of Venus [44]. And dry crust would imply that mantle would also be dry. So we assume that both the crust and mantle of Venus are dry.

The temperature distribution in Venus is not known mainly because the nature of heat transfer and the degree to which radioactive elements are segregated

into the crust are unknown [45,46]. We use the thermal model by Nimmo and McKenzie [45] that has relatively small temperature gradient (~6 K/km) implying that Venusian mantle is currently warming up (for Earth, we use an oceanic geotherm corresponding to the age of 60 Myr).

The temperature in Venus is likely time dependent. At around ~500 Myr ago, there was a catastrophic over-turn of materials (cold materials transported to the interior, hot materials to the surface), after which Venus has been geologically quiet. During this quiet time, the near surface layers cooled down while the interior warmed up. The deformation of the Venusian crust likely occurred in a variety of stages corresponding to different thermal structures [43,47]. Most of tectonic features showing deformation of Venus are with short-wavelength (~10s km) suggesting the shallow origin. It is likely that those events occurred soon after the large scale over-turn where thermal gradient was high ($dT/dz > 15$ K/km) [47].

In this paper, we are concerned with the long-wavelength tectonics such as (the absence of) plate boundaries in the stable stage after the catastrophic over-turn, and therefore we use the temperature profile with relatively low thermal gradients (dT/dz)=6 K/km corresponds to the Nimmo-McKenzie model [45].

Water in the oceanic lithosphere

The oceanic lithosphere is formed at mid-ocean ridges as a residue of partial melting. Consequently, it is essentially dry [48], i.e., depleted with incompatible elements including hydrogen (water). Indeed, the water content in minerals in the oceanic lithosphere is much less than the saturation limit [49,50], indicating a low water fugacity.

The hydrothermal activity near mid-ocean ridges hydrates the shallow regions of the oceanic lithosphere. The depth extent to which this hydration occurs can be estimated from the direct sampling by deep ocean drilling [51] and/or using the observed heat flow patterns [52]. Both approaches suggest a depth extent of hydrothermal alteration to ~5 km.

Various models were proposed to suggest deeper hydration down to ~20 km depth or more [53-57]. Some proposed that normal faulting near the trench might bring water into the oceanic lithosphere [53,54]. However, for most of earthquakes in the oceanic lithosphere, including normal fault earthquakes near the trench, fracture starts in the interior of the lithosphere and propagates to the surface and to the deeper region [58,59]. Therefore it is not clear how water penetrates into the lithosphere, particularly in the deep region where stress state is compressional. In addition, the amount and the type of hydrous minerals formed by such possible deep hydration are unknown (serpentine does not reduce the friction coefficient so much [60] although talc does [61]). Finally, the presence of metastable olivine in the deep Mariana slab suggests that a majority of the slab is dry: otherwise metastable olivine would not survive [62,63].

Dynamics of and stress evolution in a heterogeneous fault

The large reduction in friction coefficient by thermal weakening provides an explanation for why the lithosphere on Earth is weak but the lithosphere on Venus is strong. Also, the large contrast between static and dynamic friction also explains the puzzling observation of large difference between the magnitude of stress drops (~10 MPa or less [64]) and the magnitude of tectonic stress measured by the *in-situ* stress measurements (~100 MPa [65]).

However, such a model contains some subtleties. For example, if the initial strength corresponds to static friction exceeds tectonic stress on the lithosphere, how does a fault motion starts that would lead to thermal weakening? We believe that a key to address this question is a concept that the stress in the lithosphere is heterogeneous caused by the mechanical heterogeneity such as the presence of strong regions (asperities) surrounded by relatively weak regions [66]. Such a concept has strong support from the observations on the source processes of earthquakes [67,68] as well as the statistical nature of seismic activities, e.g., the Gutenberg-Richter (or the Ishimoto-Iida) law [9,69]. In these cases, even if the average stress is lower than the critical stress corresponding to the static friction, there will be regions where the stress is higher than the average stress, and fault motion could start in these regions when the local stress reaches the critical stress needed to overcome static friction.

We consider a fault in which strong asperities are present in the weak background (**Fig. 6**). Weak regions may be the regions with small asperities or the fault plane covered by a soft material. Strong regions may correspond to large

asperities (as will be shown below, a large asperity is more difficult to break than a small one). In this simplified model, slip or creep in the weak regions leads to the stress concentration in the large asperities (strong regions), and the stress in the large asperities increases with time. When the stress in the large asperities reaches a critical value, they will break. This will release the stress there, and from that time on, a similar sequence of stress evolution will occur (e.g., [70,71]).

The above process can be translated into a semi-quantitative model as follows. Deformation of a large asperity is initially elastic, and the elastic strain in the asperity is produced by deformation of surrounding weak regions. In such a case stress at the asperity (σ_{asp}) is related to strain (ε) as $\sigma_{asp} = C \cdot \varepsilon$ where *C* is the elastic modulus of the asperity. The strain in an asperity is the ratio of the displacement and the characteristic dimension of an asperity in which deformation occurs. We assume that the characteristic dimension is the same as the size of the asperity, L_{asp}. Then the elastic strain of the asperity is given by $\varepsilon = \frac{\upsilon \cdot t}{L_{asp}}$ where υ is the average velocity of fault motion which is proportional to the velocity of plate motion, $\upsilon = \xi \cdot \upsilon_{plate}$, where $\xi (\leq 1)$ is a constant related to the orientation of slip relative to the relative plate motion and t is the time (measured after the last failure of the asperity). Therefore the stress on a large asperity (σ_{peak}) evolves with time as,

$$\sigma_{asp} = C \frac{\xi \cdot \upsilon_{plate} \cdot t}{L_{asp}}. \qquad \text{(S-10)}$$

When this stress reaches the threshold stress to overcome static friction, fault motion starts, and the stress will be reduced. Using $\sigma_{asp} = 300$ MPa, $\upsilon_{plate} = 10^{-9}$ m/s (3 cm/y), C=100 GPa [72], L_{asp} =100 km, the local stress on a large asperity reaches the threshold stress to overcome static friction after $\sim 10^4$ years assuming $\xi = 1$. This agrees with the observations on seismicity of near trench earthquakes [73]. The relation (S-10) implies that a large asperity is more difficult to break than a small one because elastic stress due to slip (or creep) in the surrounding regions grows more slowly for a large asperity than for a small one. Consequently large earthquakes occur less frequently than small ones.

However, a model summarized by equation (S-10) contains a few simplifying assumptions. One of which is the assumption that stress evolution is *quasi-static*: deformation in the weak region occurs by creep and deformation in the strong region is elastic until threshold stress is reached. In this case, stress evolution at a large asperity is (approximately) linear with time, and one would expect a large stress drop. If deformation in the weak region involves a number of small unstable slips (small earthquakes), then *dynamic interactions* will play a role that would likely reduce the threshold (initial) stress at which a strong asperity will yield, and hence reduces the stress drop as is shown below[74,75].

A key in the dynamic interaction is the stress concentration at a propagating crack tip. Yamashita [75] investigated the dynamics of faulting in a region with distributed strength. He assumes that slip in the weak regions occur by small-scale unstable fault (crack) propagation. In such a case, stress concentration at the tip of propagating small faults enhances the stress at a large asperity. As a small fault

(crack) gets close to a large asperity, stress on the large asperity increases rapidly with time until the local stress reaches the threshold stress against static friction (σ_{peak}) (**Fig. S2**) (a more detailed study was made by Noda et al.[74]).

In this model, a large asperity will be broken when the initial stress reaches $\sigma_{initial} = \frac{\sigma_{asp}}{\zeta} = \frac{\sigma_{peak}}{\zeta}$ ($\zeta\,(>1)$ represents the role of stress concentration) rather than $\sigma_{initial} = \sigma_{peak}$. This stress amplification occurs only when the tip of a small fault approaches close to a large asperity. In these cases, the stress at a large asperity is near the peak stress only for small displacement, and hence the peak stress does not contribute to the seismic moment very much. Therefore, the stress drop observed by seismology is not $\sigma_{peak} - \sigma_{final}$ but $\Delta\sigma = \sigma_{initial} - \sigma_{final} = \frac{\sigma_{peak}}{\zeta} - \sigma_{final}$ [69,75]. This explains relatively small stress drops (less than ~10 MPa) [64,76] for earthquakes compared to $\sigma_{peak} - \sigma_{final}$. In this case, the recurrence time of earthquakes will also be modified.

To understand how such a model works, we performed a numerical simulation of stress evolution of a heterogeneous fault (**Fig. S3**). A 60º dipping normal fault is loaded at a constant rate of 10^{-9} m/s with unstable velocity-weakening properties down to 20 km depth. This contains a weak region with a static friction coefficient of 0.2 sandwiched by two strong regions with a static friction coefficient of 0.6. Both regions are assumed to follow velocity-weakening behavior. Following a standard formulation, we characterize the state-and-rate friction by two parameters "a" and "b" (see, e.g., [9]), and a-b=-3x10^{-2} for a strong

region with strong dynamic weakening, while a-b= $-4x10^{-3}$ for weak regions with normal dynamic weakening.

We simulate the evolution of fault slip using the boundary integral method with the radiation approximation (e.g., [77-79]). As we neglect the radiation of seismic waves, our estimates of strong weakening are conservative [80]. The evolution of stress on such a fault is shown in **Fig. S3**. The juxtaposition of fault areas with different stress drops creates a complex seismic cycle with full and partial ruptures of the velocity-weakening region. While the fault system is locked most of the time, most of the slip in fact occurs at high slip speeds (**Fig. S3a,c** and **S3e,g**). The stress evolution on the weak patch with normal dynamic weakening is for reference: the dynamic range of stress throughout the seismic cycle is within a few percents of the static strength and the role of earthquake dynamics does not significantly alter estimates of fault strength (**Fig. S3b,d** and **Fig. S3f,h**). In the strong patches with strong dynamic weakening, the dynamic range of stress is large, on the order of 200 MPa, but the static stress drops are low, of the order of 10 MPa, comparable to natural earthquakes. While the fault rests in quiescence at high friction (0.5) most of the time, the slip-averaged friction (just above 0.1) approaches the value of the weak patch. With this choice of parameters, the evolution of stress in such a fault is characterized by substantially higher time-averaged stress than the slip-averaged stress. The fault *in-situ* stress measurements correspond to the time-averaged stress. The low overall strength of the oceanic lithosphere is in turn explained by the low slip-averaged stress.

This model provides a reasonable explanation for how the asperities with the high static friction coefficient can be broken at a small overall stress as well as how often these events (earthquakes) could occur, and is consistent with the statistical nature of earthquakes. This model also explains the relatively small stress drops observed for the intra-plate earthquakes despite the high static friction of rocks in the laboratory.

However, details of the interaction between faults with a variety of asperity sizes are not fully understood, including the magnitude of the stress amplification factor, $\zeta = \frac{\sigma_{peak}}{\sigma_{initial}}$, and its dependence on the nature of small-scale faulting. This problem is also related to the long-standing issue of discrepancy between the tectonic stress estimated from the *in-situ* stress measurements [65] and the stress inferred from stress drop [64,81] (or stress inferred from geodynamic modeling) as well as the interpretation of the "heat-flow paradox" [82,83]. This problem deserves further studies (e.g., [71,74]).

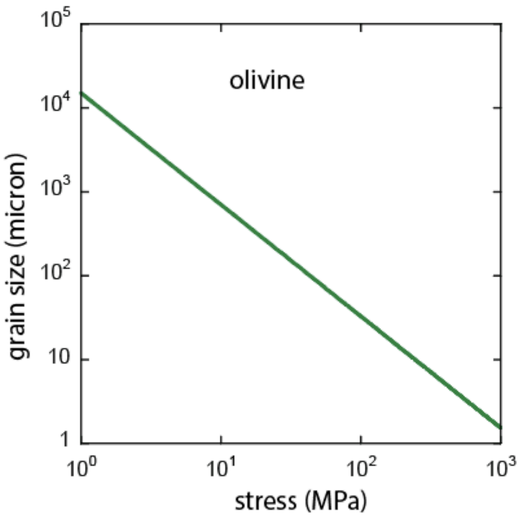

Fig. S1 Grain size versus stress relationship for olivine corresponding to dynamic recrystallization [19,21]

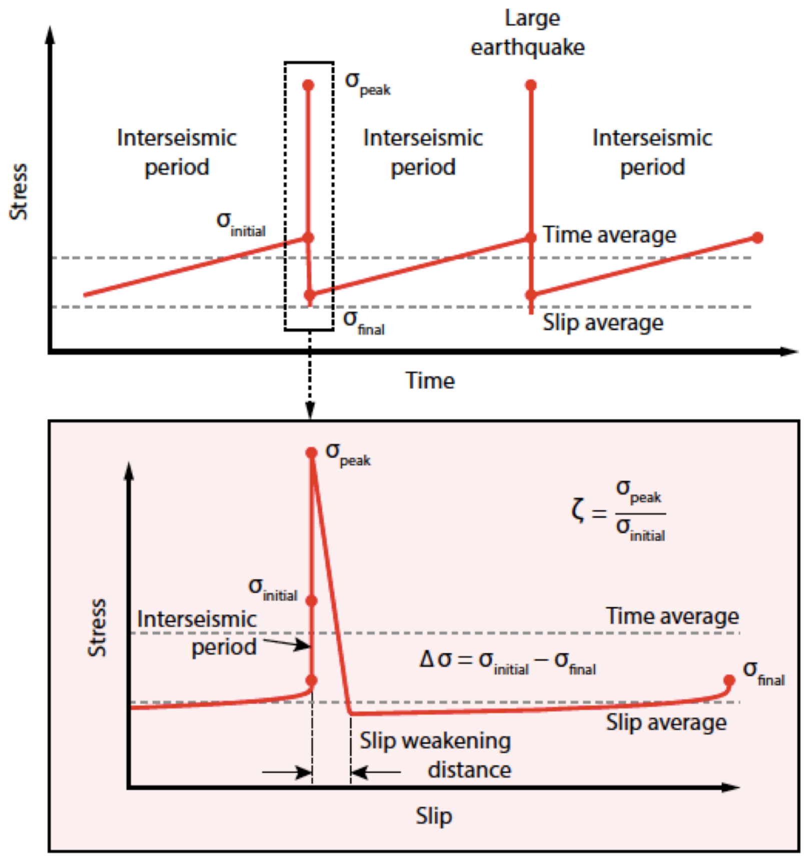

Fig. S2 Stress evolution on a heterogeneous fault with weak regions and strong large asperities (**Fig. 6a,** see also **Fig. S3**)

Due to the stress accumulation caused by slip (or creep) in the weak region, stress increases at a large asperity. When the local stress at an asperity reaches a critical value, faulting (a large earthquake) occurs. If slip in the weak region occurs through small-scale unstable slip (crack propagation), then stress evolution on a large asperity is complex (see the inset; after Yamashita [75] (see also Noda et al.[74])): When the tip of a small fault approaches a large asperity, stress increases from $\sigma_{initial}$ to σ_{peak} rapidly due to the stress concentration at the tip of the propagating crack. The stress drop observed by seismology is $\Delta\sigma = \sigma_{initial} - \sigma_{final}$ [69,75] that is much smaller than $\sigma_{peak} - \sigma_{final}$.

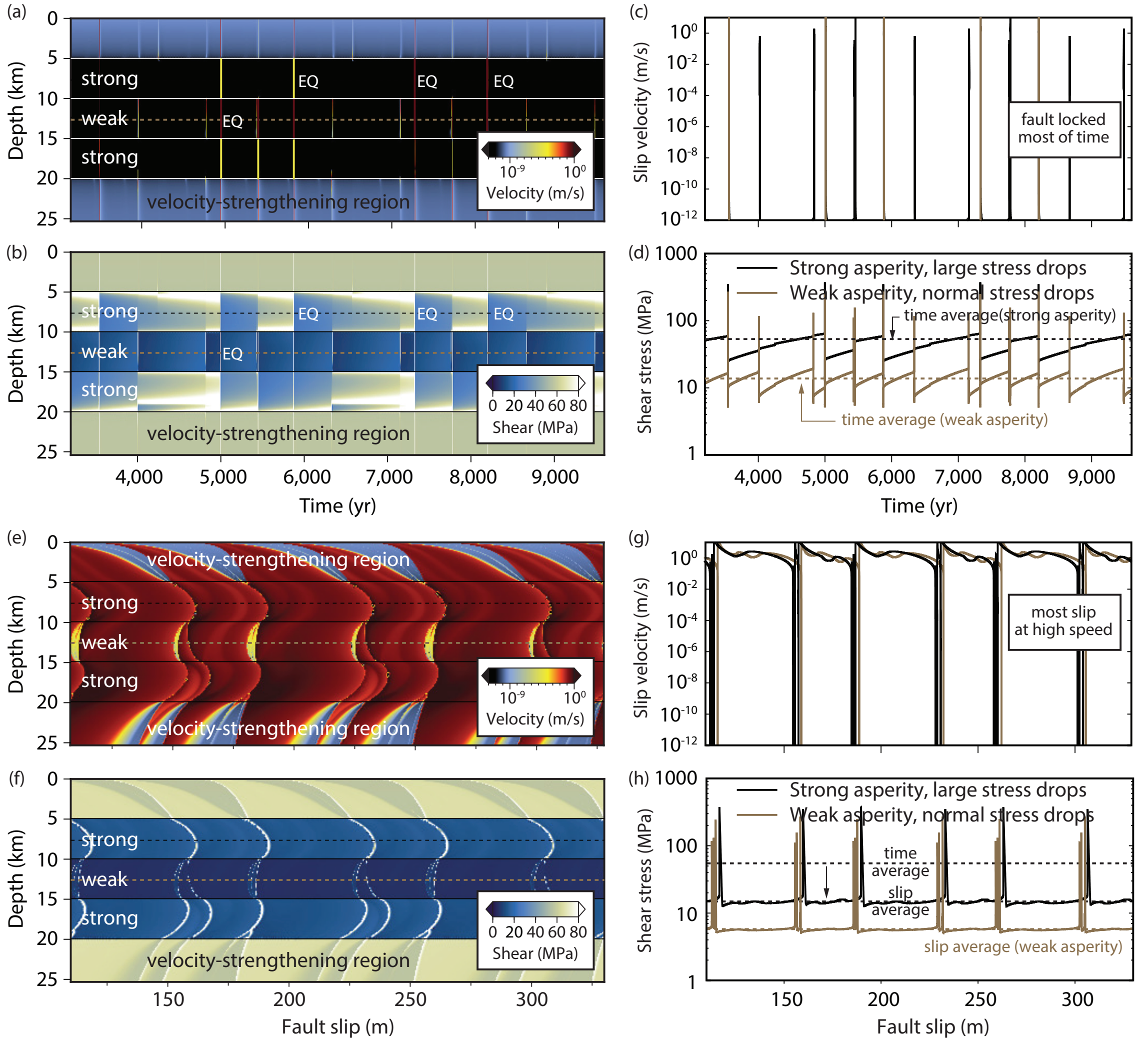
(a)
Depth (km)
strong
weak
strong
EQ
velocity-strengthening region
Velocity (m/s)
(b)
Shear (MPa)
0 20 40 60 80
Time (yr)
4,000 5,000 6,000 7,000 8,000 9,000
(c)
Slip velocity (m/s)
fault locked most of time
(d)
Shear stress (MPa)
Strong asperity, large stress drops
Weak asperity, normal stress drops
time average(strong asperity)
time average (weak asperity)
(e)
(f)
Fault slip (m)
150 200 250 300
(g)
most slip at high speed
(h)
time average
slip average
slip average (weak asperity)

Fig. S3 Evolution of slip and stress in a fault containing regions of heterogeneous strength

A 60º dipping normal fault is loaded at a constant rate of 10^{-9} m/s with unstable velocity-weakening properties down to 20 km depth.
a) Slip velocity as a function of time and depth. The fault is locked most of the time.
b) Stress as a function of time and depth. The stress in strong regions is high (60 MPa) and low (10 MPa) in weak regions most of the time, in accordance with the respective coefficients of friction under a confining pressure of 100 MPa.
c) Time series of slip velocity at the center of weak and strong asperities marked by the dashed profiles in panels a) and b).
d) Time series of shear stress at the center of weak and strong asperities.
e) Fault-slip velocity evolution as a function of cumulative fault slip and depth. Warm colors indicate typical seismic slip speeds.
f) Shear stress evolution as a function of cumulative slip and depth. Shear stress is high only during initiation of seismic episodes; otherwise, most slip occurs at low stress either in weak or strong regions.
g) Time series of slip velocity at the center of strong and weak asperities. Most of the fault slip occurs at high speed.
h) Time series of stress at the center of strong and weak asperities. The slip-averaged stress (15 MPa) is about four times less than the time-averaged stress (60MPa) in the strong regions with strong dynamic weakening.

Table S1. Flow laws used in constructing **Fig. 3** and **Fig. 5**

**deformation mechanism**	**flow law** (1)	**references, notes**
Diffusion creep	$\dot{\varepsilon} = A_1 \sigma L^{-m} \, exp\left(-\frac{E_1^* + PV_1^*}{RT}\right)$	[84], note (2)
Power-law dislocation creep	$\dot{\varepsilon} = A_2 \sigma^n \, exp\left(-\frac{E_2^* + PV_2^*}{RT}\right)$	44,85,86
Peierls mechanism	$\dot{\varepsilon} = A_3 \sigma^2 \, exp\left[-\frac{\left(E_3^* + PV_3^*\right)\left\{1-\left(\frac{\sigma}{\sigma_P}\right)^q\right\}^s}{RT}\right]$	87

(1) $\dot{\varepsilon}$: strain-rate, $A_{1,2,3}$: constants, σ : differential stress ($\sigma_1 - \sigma_3$), *L*: grain size, *n, m, q, r*: constants, $E^*_{1,2,3}$: activation energy, $V^*_{1,2,3}$: activation volume, *P*: pressure, *T*: temperature, *R*: the gas constant, σ_P : the Peierls stress

(2) The activation volume for diffusion creep has not been well constrained. We assume a tentative value of 5 cc/mol (this does not affect the results compared to the uncertainties of grain size).

1 Poirier, J.-P. *Creep of Crystals*. (Cambridge University Press, 1985).
2 Karato, S. *Deformation of Earth Materials: Introduction to the Rheology of the Solid Earth*. (Cambridge University Press, 2008).
3 Kumamoto, K. M. *et al.* Size effects resolve discrepancies in 40 years of work on low-temperature plasticity. *Science Advances* **3**, e1701338 (2017).
4 Druiventak, A., Trepmann, C. A., Renner, J. & Hanke, K. Low-temperature plasticity of olivine during high stress deformation of peridotites at lithospheric conditions - An experimental study. *Earth and Planetary Science Letters* **311**, 199-211 (2011).
5 Evans, B. & Goetze, C. Temperature variation of hardness of olivine and its implication for polycrystalline yield stress. *Journal of Geophysical Research* **84**, 5505-5524 (1979).
6 Eshelby, J. D. The forces on an elastic singularity. *Transaction of the Royal Soceity of London* **A244**, 87-112 (1951).
7 Jain, C., Korenaga, J. & Karato, S. On the yield strength of oceanic lithosphere. *Geophysical Research Letters* **44**, 10.1002/2017GL075043 (2017).
8 Paterson, M. S. & Wong, T.-F. *Experimental Rock Deformation - The Brittle Field*. (Springer, 2005).
9 Scholz, C. H. *The Mechanics of Earthquake and Faulting*. second edn, (Cambridge University of Press, 2002).
10 Jeager, J. C. *Elasticity, Fracture and Flow: With Engineering and Geological Applications*. (John Wiley & Sons, 1964).

11 Byerlee, J. D. Friction of rocks. *Pure and Applied Geophysics* **116**, 615-626 (1978).

12 Di Toro, G. *et al.* Fault lubrication during earthquakes. *Nature* **471**, 494-498 (2011).

13 Handy, M. R. Deformation regimes and the rheological evolution of fault zones in the lithosphere: the effects of pressure, temperature, grain size and time. *Tectonophysics* **163**, 119-152 (1989).

14 Jin, D., Karato, S. & Obata, M. Mechanisms of shear localization in the continental lithosphere: inference from the deformation microstructures of peridotites from the Ivrea zone, northern Italy. *Journal of Structural Geology* **20**, 195-209 (1998).

15 Bercovici, D. & Ricard, Y. Mechanisms for the generation of plate tectonics by two-phase grain-damage and pinning. *Physics of the Earth and Planetary Interiors* **202-203**, 27-55 (2012).

16 Foley, B. J., Bercovici, D. & Landuyt, W. The conditions for plate tectonics on super-Earths: Inference from convection models with damage. *Earth and Planetary Science Letters* **331-332**, 281-290 (2012).

17 Farla, R. J. M., Karato, S. & Cai, Z. Role of orthopyroxene in rheological weakening of lithosphere via dynamic recrystallization. *Proceedings of the National Academy of Sciences of the United States* **110**, 16355-16360 (2013).

18 Linckens, J., Herwegh, M., Müntener, O. & Mercolli, I. Evolution of a polymineralic mantle shear zone and the role of second phases in the localization of deformation. *Journal of Geophysical Research* **116**, 10.1029/2010JB008119 (2011).

19 Karato, S., Toriumi, M. & Fujii, T. Dynamic recrystallization of olivine single crystals during high temperature creep. *Geophysical Research Letters* **7**, 649-652 (1980).

20 Drury, M. R. in *Deformation Mechanisms, Rheology and Tectonics: From Minerals to the Lithosphere* (eds D. Gapais, J.P. Brun, & P.R. Cobbold) 143-158 (Geological Society of London, 2005).

21 van der Wal, D., Chopra, P. N., Drury, M. & Fitz Gerald, J. D. Relationships between dynamically recrystallized grain size and deformation conditions in experimentally deformed olivine rocks. *Geophysical Research Letters* **20**, 1479-1482 (1993).

22 Davies, G. F. Mechanics of subducted lithosphere. *Journal of Geophysical Research* **85**, 6304-6318 (1980).

23 Nicolas, A. Stress estimates from structural studies in some mantle peridotites. *Philosophical Transaction of Royal Society of London* **A288**, 49-57 (1978).

24 Mercier, J.-C. C. Magnitude of the continental lithospheric stresses inferred from rheomorphic petrology. *Journal of Geophysical Research* **85**, 6293-6303 (1980).

25 Karato, S. Grain-size distribution and rheology of the upper mantle. *Tectonophysics* **104**, 155-176 (1984).

26 Avé Lallemant, H. G., Mercier, J.-C. C. & Carter, N. L. Rheology of the upper mantle: inference from peridotite xenoliths. *Tectonophysics* **70**, 85-114 (1980).

27 White, S. H. Grain and sub-grain size variations across a mylonite shear zone. *Contributions to Mineralogy and Petrology* **70**, 193-202 (1979).

28 Bina, C. B. Phase transition buoyancy contributions to stresses in subducting lithosphere. *Geophysical Research Letters* **23**, 3563-3566 (1996).

29 Goto, K., Suzuki, Z. & Hamaguchi, H. Stress distribution due to olivine-spinel phase transition in descending plate and deep focus earthquakes. *Journal of Geophysical Research* **92**, 13811-13820 (1987).

30 Obata, M. & Karato, S. Ultramafic pseudotachylyte from Balmuccia peridotite, Ivrea-Verbana zone, northern Italy. *Tectonophysics* **242**, 313-328 (1995).

31 Tsutsumi, A. & Shimamoto, T. High-velocity frictional properties of gabbro. *Geophysical Research Letters* **24**, 699-702 (1997).

32 Beroza, G. C. & Ide, S. Slow earthquakes and nonvolcanic tremor. *Annual Review of Earth and Planetary Sciences* **39**, 271-296 (2011).

33 Niemeijer, A. R., Di Toro, G., Nielsen, S. & Di Felice, F. Frictional melting of gabbro under extreme experimental conditions of normal stress, acceleration, and sliding velocity. *Journal of Geophysical Research* **116**, 10.1029/2010JB008181 (2011).

34 Nielsen, H., Di Toro, G., Hirose, T. & Shimamoto, T. Frictional melt and seismic slip. *Journal of Geophysical Research* **113**, 10.1029/2007JB005122 (2008).

35 Takahashi, E. Melting of a dry peridotite KLB-1 up to14 GPa: implications on the origin of peridotitic upper mantle. *Journal of Geophysical Research* **91**, 9367-9382 (1986).

36 Rice, J. R. Heating and weakening of faults during earthquake slip. *Journal of Geophysical Research* **111**, 10.1029/2005JB004006 (2006).

37 Scholz, C. H. The brittle-plastic transition and the depth of seismic faulting. *Geol. Runds.* **77**, 319-328 (1988).

38 Kanamori, H. Seismological evidence for a lithospheric normal faulting - The Sanriku earthquake of 1933. *Physics of the Earth and Planetary Interiors* **4**, 289-300 (1971).

39 Schubert, G. *et al.* Structure and circulation of the Venus atmosphere. *Journal of Geophysical Research* **85**, 8007-8025 (1980).

40 Basilevsky, A. T. & Head, J. W. The surface of Venus. *Report on Progress in Physics* **66**, 1699-1734 (2003).

41 Donahue, T. M., Hoffman, J. H., Hodges, R. R., Jr. & Watson, A. J. Venus was wet: a measurement of the ratio of deutrium to hydrogen. *Science* **216**, 630-633 (1982).

42 Surkov, Y. A. *et al.* Venus rock composition at the Vega 2 landing site. *Journal of Geophysical Research* **91**, E215-E218 (1986).

43 Head, J. W. Processes of crustal formation and evolution on Venus: An analysis of topography, hypsometry, and crustal thickness variation. *Earth, Moon, and Planets* **50/51**, 25-55 (1990).

44 Mackwell, S. J., Zimmerman, M. E. & Kohlstedt, D. L. High-temperature deformation of dry diabase with application to tectonics on Venus. *Journal of Geophysical Research* **103**, 975-984 (1998).
45 Nimmo, F. & McKenzie, D. Volcanism and tectonics on Venus. *Annual Review of Earth and Planetary Sciences* **26**, 23-51 (1998).
46 Turcotte, D. L. How does Venus lose heat? *Journal of Geophysical Research* **100**, 16931-16940 (1995).
47 Brown, C. D. & Grimm, R. E. Recent tectonic and lithospheric thermal evolution in Venus. *Icarus* **139**, 40-48 (1999).
48 Plank, T. & Langmuir, A. H. Effects of melting regime on the composition of the oceanic crust. *Journal of Geophysical Research* **97**, 19749-19770 (1992).
49 Warren, J. M. & Hauri, E. H. Pyroxenes as tracers of mantle water variations. *Journal of Geophysical Research* **119**, 1851-1881 (2014).
50 Peslier, A. H. & Bizimis, M. Water in Hawaiian peridotites: A case for a dry metasomatized oceanic mantle lithosphere. *Geochemistry, Geophysics, Geosystems* **16**, 1211-1232 (2015).
51 Alt, J. C., Honnorez, J., Laverne, C. & Emmermann, R. Hydrothermal alteration of a 1 km section through the upper oceanic crust, deep sea drilling project Hole 504B: Mineralogy,chemistry, and evolution of seawater-basalt interactions. *Journal of Geophysical Research* **91**, 10,309-310,335 (1986).
52 Fehn, U., Green, K. E., Von Herzen, R. P. & Cathles, L. M. Numerical models for the hydrothermal field at the Galapagos Spreading Center. *Journal of Geophysical Research* **88**, 1033-1048 (1983).
53 Garth, T. & Rietbrock, A. Order of magnitude increase in subducted H_2O due to hydrated normal faults within the Wadati-Benioff zone. *Geology* **42**, 207-210 (2014).
54 Faccenda, M., Gerya, T. V. & Burlini, L. Deep slab hydration induced by bending-related variations in tectonic pressure. *Nature Geoscience* **2**, 790-793 (2009).
55 Faccenda, M., Gerya, T. V., Mancktelow, N. S. & Moresi, L. Fluid flow during slab bending and dehydration: Implications for intermediate-depth seismicity, slab weakening and deep water cycling. *Geochemistry, Geophysics, Geosystems* **13**, 10.1029/2011GC003860 (2012).
56 Korenaga, J. Thermal cracking and the deep hydration of oceanic lithosphere: A key to the generation of plate tectonics? *Journal of Geophysical Research* **112**, 10.1029/2006JB004502 (2007).
57 Seno, T. & Yamanaka, Y. in *Subduction: Top to Bottom* (eds G.E. Bebout, D.W. Scholl, S.H. Kirby, & J.P. Platt) 347-355 (American Geophysical Union, 1996).
58 Seno, T. & Honda, S. Depth extent analysis of the 1981 October 17 Chile earthquake. *Bulletin of Earthquake Research Institute* **65**, 1-32 (1990).
59 Forsyth, D. W. Determinations of focal depths of earthquakes associated with the bending of oceanic plates at trenches. *Physics of the Earth and Planetary Interiors* **28**, 141-160 (1992).
60 Chernak, L. J. & Hirth, G. Deformation of antigorite serpentine at high temperature and pressure. *Earth and Planetary Science Letters* **296**, 23-33 (2010).

61 Hirauchi, K.-i., Fukushima, K., Kido, M., Muto, J. & Okamoto, A. Reaction-induced rheological weakening enables oceanic plate subduction. *Nature Communications* **7**, 12550 (2016).

62 Kaneshima, S., Okamoto, T. & Takenaka, H. Evidence for a metastable olivine wedge inside the subducted Mariana slab. *Earth and Planetary Science Letters* **258**, 219-227 (2007).

63 Kubo, T., Kaneshima, S., Torii, Y. & Yoshioka, S. Seismological and experimental constraints on metastable phase transformations and rheology of the Mariana slab. *Earth and Planetary Science Letters* **287**, 12-23 (2009).

64 Kanamori, H. & Anderson, D. L. Theoretical basis of some empirical relations in seismology. *Bulletin of Seismological Soceity of America* **65**, 1073-1095 (1975).

65 Zoback, M. D. & Townend, J. Implications of hydrostatic pore pressures and high crustal strength for the deformation of intraplate lithosphere. *Tectonophysics* **336**, 19-30 (2001).

66 Lay, T., Kanamori, H. & Ruff, L. The asperity model and the nature of large subduction zone earthquakes. *Earthquake Prediction Research* **1**, 3-71 (1982).

67 Lay, T. & Kanamori, H. in *Earthquake Prediction* (eds D.W. Simpson & P.G. Richards) 579-592 (1981).

68 Aki, K. Asperities, barriers, characteristic earthquakes, and strong motion prediction. *Journal of Geophysical Research* **89**, 5867-5872 (1984).

69 Lay, T. & Wallace, T. C. *Modern Global Seismology*. (Academic Press, 1995).

70 Barbot, S., Lapusta, N. & Avouac, J.-P. Under the hood of the eathqukae machine: Toward predictive modeling of the seismic cycle. *Science* **336**, 707-710 (2012).

71 van Dinther, Y., Mai, P. M., Dalguer, L. A. & Gerya, T. V. Modeling the seismic cycle in subduction zones: The role and spatiotemporal occurrence of off-megathrust earthquakes. *Geophysical Research Letters* **41**, 1194-1201 (2014).

72 Bass, J. D. in *Mineral Physics and Crystallography: A Handbook of Physical Constants* (ed T.J. Ahrens) 46-63 (American Geophysical Union, 1995).

73 Chapple, W. M. & Forsyth, D. W. Earthquakes and bending of plates at trenches. *Journal of Geophysical Research* **84**, 6729-6749 (1979).

74 Noda, H., Dunham, E. M. & Rice, J. R. Earthquake ruptures with thermal weakening and the operation of major faults at low overall stress levels. *Journal of Geophysical Research* **114**, 10.1029/2008JB006143 (2009).

75 Yamashita, T. On the dynamical process of fault motion in the presence of friction and inhomogeneous initial stress Part I. Rupture propagation. *Journal of Physics of the Earth* **24**, 417-444 (1976).

76 Allmann, B. P. & Shearer, P. M. Global variations of stress drop for moderate to large earthquakes. *Journal of Geophysical Research* **114**, 10.1029/2008JB005821 (2009).

77 Qiu, Q. *et al.* The mechanism of partial rupture of a locked megathrust: The role of fault morphology. *Geology* **44**, 875-878 (2016).

78 Goswami, A. & Barbot, S. Slow-slip events in semi-brittle serpentine zones. *Scientific Reports* **8**, 6181 (2018).

79 Barbot, S. Asthenosphere fow modulated by megathrust earthquake cycle. *Geophysical Research Letters* in press (2018).

80 Thomas, M. Y., Lapusta, N., Noda, H. & Avouac, J.-P. Quasi-dynamic versus fully dynamic simulations of earthquakes and aseismic slip with and without enhanced coseismic weakening. *Journal of Geophysical Research* **119**, 1986-2004 (2014).

81 Kanamori, H. Determination of tectonic stress associated with earthquake faulting, the Tottori earthquake of 1943. *Physics of the Earth and Planetary Interiors* **5**, 426-434 (1972).

82 Lachenbruch, A. & Sass, J. Heat flow and energetics of the San Andreas fault zone. *Journal of Geophysical Research* **85**, 6185-6222 (1980).

83 Lachenbruch, A. H. & Sass, J. H. Heat flow from Cajon Pass, fault strength, and tectonic implications. *Journal of Geophysical Research* **97**, 4995-5015 (1992).

84 Hirth, G. & Kohlstedt, D. L. Experimental constraints on the dynamics of partially molten upper mantle: deformation in the diffusion creep regime. *Journal of Geophysical Research* **100**, 1981-2001 (1995).

85 Karato, S. & Jung, H. Effects of pressure on high-temperature dislocation creep in olivine polycrystals. *Philosophical Magazine, A.* **83**, 401-414 (2003).

86 Hirth, G. & Kohlstedt, D. L. Experimental constraints on the dynamics of partially molten upper mantle: deformation in the dislocation creep regime. *Journal of Geophysical Research* **100**, 15441-15450 (1995).

87 Mei, S., Suzuki, A. M., Kohlstedt, D. L., Dixon, N. A. & Durham, W. B. Experimental constraints on the strength of the lithospheric mantle. *Journal of Geophysical Research* **115**, 10.1029/2009JB006873 (2010).
